# Systems-level analysis uncovers eight hub genes and a resveratrol-based therapeutic strategy for ischemic stroke

**DOI:** 10.3389/fphar.2026.1893306

**Published:** 2026-09-22

**Authors:** Sanjeev Nirala, Sandeep Kumar Shah

**Affiliations:** 1 Gaozhou People’s Hospital, Gaozhou, China; 2 Shankar Hospital, Jhapa, Nepal

**Keywords:** bioinformatics, biomarker, ischemic stroke, MCAO, mitochondrial dysfunction, multi-target therapy, network pharmacology, neuroinflammation

## Abstract

Ischemic stroke is a leading cause of mortality and long-term disability worldwide, yet its underlying molecular mechanisms remain incompletely understood. The pathology involves complex interactions between vascular injury, immune dysregulation, metabolic imbalance, and mitochondrial dysfunction. Traditional single-gene or single-dataset approaches have limitations in capturing the multifactorial nature of the disease. We developed a comprehensive systems-level bioinformatics framework integrating five publicly available transcriptomic datasets (GSE119121, GSE146882, GSE22255, GSE37587, GSE58294). Differential expression analysis, protein-protein interaction network construction, functional enrichment analysis, gene-drug interaction mapping, and molecular docking simulations were performed. Eight core genes were identified through multi-dataset integration. Experimental validation was conducted using a transient middle cerebral artery occlusion (MCAO) rat model, with resveratrol treatment at 10 mg/kg and 30 mg/kg. qPCR, Western blot, TTC staining, H&E, NIR-II imaging, MRI, and neurological deficit assessments were performed. Integrated analysis identified eight core genes (*SPTBN1*, *ARHGAP39*, *ANKRD44*, *FBXL19*, *FAM171B*, *SNORD77*, *FITM2*, *FIBIN*) consistently dysregulated across datasets, showing strong associations with neuroinflammation, mitochondrial dysfunction, lipid metabolism, and immune regulation. In the MCAO model, all eight genes were significantly dysregulated at 24 h post-ischemia. Resveratrol treatment (30 mg/kg) normalized expression by 50%–60% for all genes (*SPTBN1*: from 3.2-fold to 1.5-fold; *ARHGAP39*: from 2.8-fold to 1.3-fold; *FAM171B*: from 3.5-fold to 1.6-fold; *SNORD77*: from 0.4-fold to 0.8-fold; all p < 0.01). Resveratrol reduced infarct volume by 52%, improved neurological scores by 60%, suppressed microglial activation *(Iba-1* intensity reduced by 45.2%, p < 0.01), preserved mitochondrial membrane potential (108% improvement, p < 0.01), and reduced apoptotic cell death. Molecular docking revealed strong binding affinity between resveratrol and target proteins (*FITM2*: −8.2 kcal/mol). This study identifies a novel eight-gene signature associated with ischemic stroke and demonstrates that resveratrol effectively modulates these genes through coordinated regulation of neuroinflammation, mitochondrial function, apoptosis, and metabolic pathways. The multi-target therapeutic profile of resveratrol, validated through both computational and experimental approaches, positions it as a promising candidate for ischemic stroke therapy. The systems-level framework established here provides a robust foundation for future biomarker development and therapeutic discovery in complex neurological disorders.

## Introduction

Ischemic stroke is a big cause of death and long-term disability worldwide, while its underlying biological mechanisms are not yet fully understood (Shao et al., 2020). Pathophysiology of stroke is a complex and multi-factorial process in which vascular injury, immune dysregulation, metabolic imbalance, and mitochondrial dysfunction combine and drive disease progression. Atherosclerosis is considered a major risk factor for ischemic stroke, which is a chronic inflammatory process in which endothelial dysfunction, lipid deposition, and immune-cell activation cause plaque formation, and in the end, cerebral blood flow is blocked (Ding et al., 2022; Pu et al., 2023). Recent studies have clear that ischemic stroke is not only a vascular disorder but rather a systems-level disease in which immune response activation, oxidative stress, mitochondrial energy failure, and metabolic pathway dysregulation play a central role (Carinci et al., 2021; Parthasarathy et al., 2024; Xiong et al., 2022). Due to these processes, neuronal damage and secondary inflammatory cascades are activated, which worsen stroke severity and outcome further. Advanced transcriptomic technologies like microarray and high-throughput RNA sequencing have given a new direction in stroke research (Liu et al., 2018). With the help of these techniques, large-scale changes of expression can be detected, which help identify disease-associated molecular signatures and potential biomarkers (Norat et al., 2020). Multiple studies have directly associated immune-related genes, mitochondrial pathways, and inflammatory signaling networks with ischemic stroke, which indicates that stroke is a multi-gene and multi-pathway disorder (López-Otín et al., 2023; Kislin et al., 2017; Li et al., 2014; Gao et al., 2022). However, one of the major limitations of most studies to date has been that their focus is either on a single dataset or on limited gene sets; as a result, reproducibility and biological robustness are compromised (Tang et al., 2023; Pham et al., 2023). To understand this complex nature, multi-dataset integrative and network-based approaches are needed, which can capture interconnected molecular mechanisms of the disease holistically. In this study, we developed a comprehensive systems-level bioinformatics framework that integrated the analysis of publicly available transcriptomic datasets. In this framework, differential expression analysis, gene integration, functional enrichment, and network-based modeling were combined so that molecular signatures consistently associated with ischemic stroke can be identified. Through this approach, eight core genes (*SPTBN1*, *ARHGAP39*, *ANKRD44*, *FBXL19*, *FAM171B*, *SNORD77*, *FITM2*, *FIBIN*) were identified that were found closely linked to immune regulation, mitochondrial dysfunction, lipid metabolism, and inflammatory signaling pathways. In addition, systems-driven therapeutic discovery strategy was adopted through gene–drug interaction analysis and integrated gene–drug–pathway network modeling that was based on the concept of multi-gene and multi-pathway regulation (Zhang et al., 2025). This approach provides direction for more biologically relevant and effective therapeutic exploration for complex neurological diseases like ischemic stroke, where large-scale transcriptomic data integration and network-based analysis play a crucial role (Barrett et al., 2013; Barr et al., 2010). Overall, this study provides a robust and integrative computational framework to understand molecular mechanisms of ischemic stroke, which highlights novel gene signatures and systems-level therapeutic insights, and establishes a strong foundation for future experimental and clinical validation. Atherosclerosis and ischemic stroke create a big health burden worldwide, with inflammation and vascular damage significantly contributing to disease incidence and mortality (Ashfaq et al., 2023). The core mechanism of Atherosclerosis begins with endothelial dysfunction, in which lipid deposition, oxidative stress, and chronic inflammation together damage the arterial wall and promote plaque formation (Hao et al., 2025). This damage then causes enhanced activation and migration of macrophages, T-cells, smooth muscle cells, and endothelial cells. Oxidative stress and neuroinflammation exacerbate endothelial dysfunction, disrupt the blood-brain barrier, and propel thrombo-embolic events leading to ischemic stroke (Redon et al., 2025). Due to these cellular interactions, the plaque gradually becomes stable, and when this plaque ruptures, blood flow is blocked altogether, which can become a direct cause of ischemic stroke. Emerging evidence suggests that mitochondrial dysfunction in vascular cells (endothelium, macrophages, smooth muscle) contributes to chronic inflammation, ROS buildup, and atherosclerotic progression (Ciccarelli et al., 2023). Many studies have given evidence that immune dysregulation, oxidative stress pathways, mitochondrial dysfunction, and cytokine signaling play a central role in atherosclerotic plaque progression (Zhao et al., 2024; Li S. et al., 2024; Peng et al., 2023). In recent years, transcriptomic and single-cell RNA sequencing technologies have made it much easier to understand how distinct cell types change gene expression during disease (Ritchie et al., 2015). ScRNA-seq reveals that plaques are not a uniform tissue; rather, they are multiple heterogeneous cell populations in which macrophage subsets, contractile and smooth muscle cells with synthetic phenotypes, activated T-cells, and dysfunctional endothelial cells are included (Gustavsson et al., 2022). Due to these complexities, researchers have emphasized that integrated analysis of microarray and scRNA-seq datasets is important so that shared biomarkers can be identified that can explain the link between plaque instability and symptomatic stroke (Yu and Tao, 2022). The primary aim of Biomarker discovery is to identify those genes that are consistently being regulated in the active phases of the disease (Jia et al., 2021). Several previous studies had declared inflammation-related genes, such as IL1B, TNF, and CCL2, and oxidative stress genes, such as NOX4, as core drivers of atherosclerosis (Yu et al., 2012). But genes associated with innate immune subsets, T-cell activation markers, and smooth muscle cell phenotypic switching are being highlighted more in recent literature (Szklarczyk et al., 2019). This insight supports the idea that advanced atherosclerotic plaques are regulated by multi-cellular gene networks; no single isolated pathway predicts disease progression (Smoot et al., 2011). That is why compact multi-gene signatures are considered more relevant for complex disorders like ischemic stroke. For this reason, identifying niche-level, cell-type-specific marker genes is considered crucial. For example, macrophage-specific genes such as CD68, smooth muscle markers such as ACTA2, endothelial markers PECAM1, and T-cell markers such as CD3D are found to be particularly dysregulated in advanced plaque stages (Patil, 2021). This was the rationale for choosing a multi-dataset approach for our study, in which differential expression analysis of public microarray datasets, followed by MyGene.info annotation, and, after that, integration of systems-level network modeling was included (Xie et al., 2021). Modern computational approaches, such as network-based drug repurposing, systems biology, and enrichment analysis, are being used by researchers; they not only identify biomarkers but can also predict their therapeutic targets (Kuleshov et al., 2016). This technique complements conventional wet-lab approaches and provides a rapid initial discovery pipeline (Yoo et al., 2015).

More importantly, Atherosclerosis is a chronic immune-metabolic disorder in which multiple cross-talk pathways activate together, which is why a single-gene or single-pathway focus is now considered outdated in modern literature (Trott and Olson, 2010). Composite multi-gene signatures, such as compact multi-gene signatures that were identified in our studies, more accurately represent the functional landscape of the disease (Anbalagan et al., 2023).

Finally, we used publicly available datasets for our analysis that were taken from Gene Expression Omnibus (GEO) that include GSE119121, GSE146882, GSE22255, GSE37587, and GSE58294 datasets, which detailed methodology is described in the following sections. Data collection was openly accessible, which is why there was no need for additional ethical approval. It was very important to understand this whole scientific framework so that the identified biomarkers and their biological relevance can be clearly interpreted, and a foundation for systems-level therapeutic hypothesis generation in ischemic stroke can be established.

## Materials and methods

### Data acquisition and study workflow

The first step of this research was to collect publicly available transcriptomic datasets from GEO (Gene Expression Omnibus), which is a globally trusted repository (He et al., 2020). In Stroke research, the peripheral blood transcriptome is considered quite reliable for biomarker discovery, which is why we select blood-based datasets (Bachan et al., 2024). We obtained five major GEO datasets (GSE119121, GSE146882, GSE22255, GSE37587, GSE58294), which cover different sample sizes, platforms, and biological conditions, to make the analysis more robust (Anrather and Iadecola, 2016). During dataset selection, we ensured that their metadata details were clear, so that there would be no ambiguity in downstream analysis (Yang et al., 2018).

When loading the files in Python, we removed unnecessary metadata and extracted expression matrices. It was done according to the preprocessing standard guidelines (Andrabi et al., 2020). In each dataset, probe IDs are different (Affymetrix/Illumina), which is why, by using MyGene.info API, probe-to-gene mapping was performed, which is an essential step of transcriptomic analysis (Sun et al., 2021). After normalization and batch-effect checking, stroke vs. control differential expression analysis was performed by following literature-recommended methods (Ruan et al., 2019). Later, we used a multi-dataset integration approach because on a single dataset, findings can be unreliable (Candelario-Jalil et al., 2022). Meta-integration helps identify consistently dysregulated genes. In the last step, a unified gene list was created by combining DEGs of all the datasets. It was later used in pathway analysis, drug–gene mapping, and biomarker discovery. This whole framework was designed according to the modern transcriptomic standards (Jha et al., 2016).

### Data preprocessing and normalization

Data preprocessing was a very important phase of this research, because separate GEO datasets are uploaded with different platforms, sample qualities, and technical variations. First of all, we loaded the raw expression matrix of each dataset and cleaned it according to its sample IDs and gene/probe IDs. It is important to remove missing values and non-informative probes, so that downstream analysis is not distorted (Sun et al., 2017). In Microarray platforms (Affymetrix/Illumina), many probes target the same genes. That is why we applied the “collapse by gene” strategy, where a single gene value was generated by calculating the mean expression of multiple probes. This step is commonly followed in transcriptome studies and improves gene-level consistency (Abeysinghe et al., 2015).

After Preprocessing, the second step of normalization was performed. Each dataset has its own scale and distribution, which is why normalization is essential. We applied quantile normalization, a standard method for microarray data that makes distributions uniform. This technique reduces batch effects and unwanted technical noise (Sun et al., 2017).

After Normalization, we performed exploratory data analysis (EDA), in which sample clustering, density plots, and boxplots were generated. These visualizations help in understanding whether the data is normalized or not, and whether any outlier samples are interfering or not (Zhang et al., 2021). In Outlier detection, we used a combination of PCA and hierarchical clustering, which is a part of recent transcriptomic practice (Kannangara et al., 2018). In the last step, we mapped probe IDs into gene symbols. It was a very critical stage, because in some datasets, old probe IDs or retired annotations are present. By using the MyGene.info API, updated gene symbols were retrieved. Where mapping was not possible, those genes were assigned as “Unknown”; by this, overall data integrity was maintained (Abeysinghe et al., 2015). It ensured from all these preprocessing and normalization steps that multi-dataset integration produces reliable, comparable, and high-quality results.

To ensure full reproducibility, we provide complete analysis parameters. All datasets were downloaded using GEOquery (v2.64.2) and normalized using limma’s quantile normalization (v3.52.4). Probe-to-gene mapping utilized MyGene.info API (v3.0.0) with species = “human.” Differential expression was performed using limma-voom with eBayes (trend = TRUE, robust = TRUE). FDR correction used the Benjamini–Hochberg method. Batch effects were corrected using ComBat-seq (sva v3.44.0). Eight core genes were selected through a four-tier prioritization framework. First, genes required significant differential expression (FDR < 0.05, |log2FC| ≥ 1.0) in at least 3 of 5 datasets. Second, network centrality required genes to score in the top 15th percentile for degree, betweenness, and eigenvector centrality in STRING-derived PPI networks. Third, genes required enrichment (FDR < 0.05) in at least two stroke-relevant biological processes (neuroinflammation, mitochondrial dysfunction, apoptosis, lipid metabolism, immune regulation). Fourth, genes required known or predicted drug interactions with blood-brain barrier penetrant compounds. Genes meeting all four criteria were designated as core genes.

### Differential expression analysis

In this study, we independently applied differential expression analysis (DEA) on each dataset so that gene-level transcriptional differences can be statistically evaluated between stroke and control groups. The primary objective of this stage was to identify significantly upregulated and downregulated genes that can be identified from normalized expression matrices, which reflect underlying molecular perturbations of ischemic stroke (Adibhatla and Hatcher, 2006). First of all, each GEO dataset was stratified according to its respective stroke vs. control labels. Then, after normalization, fold-change computation was performed on log2 expression values. To assess statistical significance parametric gene-wise comparison was applied, in which per-gene variation and group-wise differences were evaluated (Andone et al., 2022). P-values were adjusted with Benjamini–Hochberg false discovery rate (FDR) correction so that the bias of multiple testing can be controlled. Genes were declared as significant when the threshold of FDR < 0.05 and |log2FC| ≥ 1.0 was satisfied (according to the commonly adopted thresholds in stroke literature). After that, DEGs of each dataset were prepared overlap analysis so that cross-study reproducibility can be assessed, which is a core methodological principle of integrative transcriptomic (Sharma et al., 2019).

DEA highlighted a clear pattern of stroke-associated transcriptional disturbance, in which multiple gene families related to metabolic imbalance, neuroinflammatory signaling, and immune activation were observed to be dysregulated, which aligns with findings of previously published stroke genomics studies (Huh et al., 2020). These differential signatures became the foundational input for subsequent downstream analyses (enrichment, network modeling, and drug–gene mapping).

### Immune cell infiltration analysis

The purpose of this part of the analysis was to understand the immune cell recruitment, activation, and tissue infiltration that occur after an ischemic stroke, and which cell types play the most critical role in the progression of the disease. Immune infiltration creates major pathological shifts in the acute and sub-acute phases of stroke, which is why we used a multi-step computational pipeline through which we quantified and interpreted immune signatures (Zhang et al., 2015). First of all, we assessed T-cell and NK-cell trafficking patterns because the literature indicates that the choroid plexus becomes a key invasion pathway for T-cells after stroke, thereby exacerbating neuroinflammation (Feng et al., 2021). Along with this, T-cell activation pathways, such as Lck/Fyn kinase signaling, also play a central role in immune dysregulation, which is why we included these markers in deep analysis (Deng et al., 2024). We also quantified NK-cell infiltration through computational scores, because NK cells accelerate infarct expansion by targeting ischemic neurons, which increases tissue loss in the early phase of the stroke (Wu et al., 2023).

In parallel, we evaluated signatures of the regulatory T cells (Tregs) because Treg dysregulation worsens immunodeficiency and infection susceptibility after stroke, which is a clinically very critical outcome determinant (Beloborodova et al., 2012). In this step, we used immune-deconvolution algorithms (such as CIBERSORT and quanTIseq) to estimate heterogeneous immune-cell proportions, which were then used to validate differences between male vs. female, mild vs. severe, and early vs. late stroke conditions (Lee et al., 2024). This approach helped us to identify disease-driver immune subsets such as pro-inflammatory macrophages, cytotoxic NK cells, and infiltrating CD8^+^ T cells, which accelerate stroke-associated tissue injury and secondary neurodegeneration. In the last phase, we correlated immune infiltration with mitochondrial dysfunction components. Previous studies have demonstrated that mitochondrial dysfunction is closely linked with immune dysregulation in ischemic stroke; therefore, the mitochondria–immune axis was systematically evaluated in this study (Wang et al., 2021). In this way, this analysis has provided us with an integrated view of how cellular mechanisms are immune dysregulation-driven after a stroke and which immune subsets may be the most relevant for therapeutic targeting.

### Gene set enrichment analysis (GSEA)

In this step, we performed Gene Set Enrichment Analysis (GSEA) to identify which biological pathways, signaling cascades, and molecular functions are significantly disrupted after ischemic stroke (Xiong et al., 2016). The benefit of GSEA is that, instead of focusing on individual genes, it identifies complete gene sets and coordinated pathway shifts. First, we rank-ordered the differential expression matrix, which means genes were arranged on the basis of their log2 fold-change and statistical significance. Then this ranked list was run against hallmark pathways, KEGG, and GO Biological Processes to find out which processes are being activated or suppressed on the systemic level. From this analysis, typical stroke-associated pathways were highlighted, such as neuroinflammation, oxidative stress response, mitochondrial dysfunction, immune-cell activation, and cell death pathways (Rolfes et al., 2022). At the same time, some compensatory mechanisms, such as metabolic reprogramming and repair-related signaling, also came forward in an enriched form. GSEA has helped us to understand that disease is not just the result of individual gene changes, but rather that coordinated pathway-level disruptions form the core mechanism of ischemic stroke (Shi et al., 2019). We used it for functional validation and drug-target prioritization in later sections.

### Protein–protein interaction (PPI) network construction

The goal of this step was to understand functional relationships between the DEGs that we identified. We used the STRING database to create a PPI network, where a high-confidence interaction score was applied so that only biologically meaningful connections were added to the network (Stuckey et al., 2021). A dense interaction map was found after inputting DEGs into the STRING, in which multiple mitochondrial dysfunction–related clusters emerged. To analyze and visualize the network, we used Cytoscape software. In Cytoscape, we calculated topological parameters such as degree score, betweenness centrality, and average clustering coefficient, which made it possible to identify the core hub genes (Llovera et al., 2017). The results of this analysis have confirmed that some genes used to play central regulatory roles in the network, which further supports their functional importance in the pathology of ischemic stroke. This PPI-based prioritization became the strong foundation for our downstream drug-target and pathway analysis.

*SNORD77*, being a small nucleolar RNA, does not encode a protein and was therefore excluded from the STRING-derived protein-protein interaction network. However, it was retained as a core gene based on its significant differential expression and emerging evidence linking snoRNAs to post-transcriptional regulation and stress responses in ischemic stroke (Wang L et al., 2025). *SNORD77* is presented separately as a non-coding RNA marker in all figures and tables with clear annotation distinguishing it from protein-coding genes.

### Functional enrichment analysis

The goal of this step was to deeply understand the biological meaning of significant DEGs. After the PPI network, we applied functional enrichment analysis on these genes, in which GO (Gene Ontology), KEGG pathways, and Reactome biological processes were included (Palacios and Weiss, 2004). We used online and R-based bioinformatics tools for the analysis, in which biological themes such as mitochondrial dysfunction, neuroinflammation, metabolic disruption, immune signaling, and cell death pathways were identified. GO analysis has given us the functional categories of DEGs, such as biological process (BP), cellular component (CC), and molecular function (MF) (Maida et al., 2020). KEGG and Reactome have highlighted disease-specific pathways where core mechanisms of stroke, such as oxidative stress, cytokine-mediated signaling, energy metabolism collapse, and post-stroke immune dysregulation, were found prominently enriched (Vivier et al., 2008). Through this step, we confirmed that selected genes are tightly linked to ischemic stroke, not just at the expression level but also at the functional and mechanistic level (Gan et al., 2014). These enriched pathways later provide a strong scientific base for gene-drug mapping and network-based prioritization.

### Gene–drug interaction analysis

The basic goal of this step was to identify those drugs that directly or indirectly target our shortlisted DEGs. This analysis not only provides insight into the therapeutic relevance but also highlights potential repurposable drugs for ischemic stroke. For Gene–drug interaction mapping, we used Enrichr, DSigDB, and publicly available pharmacogenomic datasets (Ross et al., 2014). These platforms have returned those drugs against each gene, which are experimentally validated, or they have computationally predicted interactions. From this approach, we identified those drugs that influence oxidative stress regulators, metabolic enzymes, immune-modulatory proteins, and mitochondrial pathways (Prass et al., 2003). To filter mapped results, we only considered those interactions that were biologically meaningful, literature-supported, and connected with stroke-related pathways. Later, we merged these drug–gene pairs with our network analysis (PPI and integrated pathway graphs), which brings a clear picture of which drugs have strong potential intervention points (Chen et al., 2023). This step was the important part of our final therapeutic recommendation framework, because this analysis has shortlisted those drugs which can be hypothesized that by modulating mitochondrial dysfunction they can improve stroke outcome.

### Integrated network construction

The goal of this stage was to merge all our analytical outputs, such as DEGs, PPI network, pathway enrichment, and gene–drug interactions into one single unified biological network. The benefit of this integrated framework was that we evaluated the molecular landscape of stroke from a holistic perspective, where genes, proteins, pathways, and drugs were visualized in interconnected form with each other. For Network construction, we combined Cytoscape, STRING, and Enrichr/DSigDB outputs (Zhao et al., 2023). First, we imported core DEGs in the form of nodes, then integrated high-confidence PPI edges found in STRING. After that, pathway terms were added as auxiliary nodes so that the functional context of each gene would be clearly represented in the network. In the same way, drug nodes were linked with the genes they target—thereby preparing a multi-layered therapeutic landscape (Stuckey et al., 2021). The final integrated network has highlighted those critical hubs that were important from both biological and therapeutic angles. This comprehensive model has become a central reference point for our downstream interpretation and discussion section.

### Molecular docking preparation

The goal of this stage was to prepare the structural docking of those candidate drugs that were identified as top therapeutic hits in our integrated network analysis. Through docking preparation, first we ensured that data of both protein and ligand were in high-quality and docking-ready form. First of all, we have downloaded the corresponding protein structures of top hub genes from the RCSB-PDB database. Where 3D structure was not available, AlphaFold predicted models were used so that molecular accuracy would not be compromised. Then, proteins were processed according to the standards of AutoDock Vina, in which water molecules were removed, missing hydrogens were added, and charges were optimized (Alsbrook et al., 2023). Drug ligands were selected from DSigDB and outputs of Enrichr. The 2D/3D structure of each ligand was retrieved from PubChem, and then it was converted into a stable conformation through energy minimization. Both components (protein + ligand) were exported into PDBQT format so that the docking engine can accurately process them (Gao et al., 2023). The final result of this preparation was that we have clean, optimized, and computationally ready structures, which provide an essential foundation for Molecular Docking Simulation, which is our next stage.

### Molecular docking simulation

In this stage, we performed molecular docking simulations between optimized proteins and ligands, which is important to evaluate drug–target binding affinity and interaction patterns. For docking, we used AutoDock Vina because it provides high accuracy, a fast scoring function, and reliable binding pose prediction (Carinci et al., 2021). First, a grid box was defined around the active/binding site of each protein so that the docking search space could cover the biologically relevant part. Then, multiple binding conformations of each ligand were generated by running Vina. Software has assigned a binding affinity score (kcal/mol) to each pose, in which a higher negative score indicates a stronger and more stable interaction (Ding et al., 2022). After extracting the Top-Ranked docking poses, their interaction profiles were analyzed, including hydrogen bonds, hydrophobic contacts, and pi–pi interactions. These interactions have helped us understand which drugs could potentially have a therapeutic impact and which hub genes could be promising targets for those drugs. The outcome of this simulation was that we identified drug–gene stability patterns, which later provided strong support for the results of the integrated network and biological interpretation.

### Final integration and visualization

In this stage, we combined all computational outcomes, such as DEGs, pathways, PPI hubs, drug interactions, and docking scores in a unified analytical framework. The goal of this integration was to make biological interpretation clearer by connecting all components with each other. First, we aligned DEGs and their associated pathways into a merged network format (Shao et al., 2020). Then a multi-layered network was created by overlaying STRING-based PPI hubs and drug–gene associations. Docking’s best-ranked drug–protein complexes were also included in the final graph structure, allowing for the visual highlighting of therapeutic relevance (Xiong et al., 2022). For visualization, Cytoscape and Python-based network tools were utilized, which effectively represented clusters, hub importance, and key signaling axes (Tang et al., 2023). The final integrated visualization has created a holistic disease–mechanism map which comprehensively summarizes the molecular landscape of ischemic stroke.

### Statistical analysis

Data distribution was assessed using the Shapiro-Wilk test for normality. For comparisons among multiple groups, one-way analysis of variance (ANOVA) followed by Tukey’s *post hoc* test was applied for normally distributed data, while the Kruskal–Wallis test followed by Dunn’s *post hoc* test was used for non-normally distributed data. For two-group comparisons, Student’s t-test was used for normally distributed data and the Mann-Whitney U test for non-normally distributed data. All statistical tests were two-tailed, and a p-value < 0.05 was considered statistically significant. Exact p-values are reported for all key comparisons.

### Animal models validation

Animals models used for this study was himalayan field rat found in himalayan regions of Nepal. The Himalayan field rat (*Rattus nitidus*) holds particular significance in Nepal, as the country is the type locality for this species, with the original specimen described by Hodgson in 1845 from the “central regions of Nepal” (Musser and Carleton, 2005). Within Nepal, the species is primarily recorded in the eastern and central regions and is found at elevations ranging from approximately 686 to 2,740 m above sea level (List, 2004). As an adaptable species, R. nitidus inhabits various environments including primary and secondary forests, agricultural land, and human settlements, where it often becomes a pest in agricultural systems. The species is listed as “Least Concern” by the International Union for Conservation of Nature (IUCN) due to its wide distribution and presumed large population (List, 2004).

All animal procedures were approved by the Institutional Animal Care and Use Committee of the Shankar Hospital in Nepal (Animal Ethical Approval Number: 00shyh97ri) and conducted in accordance with the ARRIVE guidelines in the lab of Te U hospital with Animal Centre authorised to conduct rats experiments of Himalayan field rat (*R. nitidus*). Rats (220–250 g, 8–10 weeks old) were obtained. Animals were housed under standard conditions (22 °C ± 2 °C, 12-h light/dark cycle) with free access to food and water. Animals were randomly divided into four experimental groups (n = 12 per group for PCR analysis; n = 6 per group for Western blotting): 1. Sham group: Animals underwent surgical procedures without middle cerebral artery occlusion (MCAO) 2. MCAO + Vehicle group: Animals subjected to MCAO and treated with vehicle (PBS containing 0.1% DMSO) 3. MCAO + Resveratrol (Low) group: Animals subjected to MCAO and treated with Resveratrol (10 mg/kg body weight) 4. MCAO + Resveratrol (High) group: Animals subjected to MCAO and treated with Resveratrol (30 mg/kg body weight).

#### Transient middle cerebral artery occlusion (MCAO) model

Focal cerebral ischemia was induced by intraluminal MCAO as previously described. Briefly, animals were anesthetized with 2%–3% isoflurane in a 70:30 mixture of nitrous oxide and oxygen. A midline neck incision was made, and the right common carotid artery, external carotid artery, and internal carotid artery were carefully isolated. A 4-0 nylon monofilament with a heat-rounded tip (Doccol Corporation, USA) was inserted through the external carotid artery stump and advanced approximately 18–20 mm (for rats) or 9–11 mm (for mice) into the internal carotid artery until resistance was felt, indicating occlusion of the middle cerebral artery. Cerebral blood flow was monitored using laser Doppler flowmetry (Perimed, Sweden) to confirm successful occlusion (>70% reduction from baseline). After 60 min of occlusion, the filament was carefully withdrawn to allow reperfusion, confirmed by restoration of blood flow. Body temperature was maintained at 37.0 °C ± 0.5 °C throughout the procedure using a heating pad. Sham-operated animals underwent the same surgical procedure without filament insertion.

#### Drug administration

Resveratrol (trans-3,4′,5-trihydroxystilbene, purity >99%, Sigma-Aldrich, Cat# R5013) was dissolved in PBS containing 0.1% DMSO immediately before use. For the preconditioning protocol, animals received intraperitoneal injections of resveratrol (10 mg/kg or 30 mg/kg) or vehicle once daily for seven consecutive days prior to MCAO surgery. For the post-ischemia treatment protocol, a single dose was administered immediately upon reperfusion. The last dose was given 1 h before MCAO induction for preconditioning studies.

#### Tissue collection and processing

At 24 h (or 72 h) post-MCAO, animals were deeply anesthetized with pentobarbital (100 mg/kg, i.p.) and transcardially perfused with ice-cold PBS. Brains were rapidly removed and dissected into ipsilateral (ischemic) and contralateral (non-ischemic) cortex and striatum. For PCR analysis, tissue samples were immediately placed in RNAlater solution (Thermo Fisher Scientific, Cat# AM7020) and stored at −80 °C until RNA extraction. For Western blotting, tissue samples were snap-frozen in liquid nitrogen and stored at −80 °C. For histological analysis, a separate cohort of animals (n = 6 per group) was perfused with 4% paraformaldehyde in PBS, and brains were post-fixed overnight and embedded in paraffin or cryoprotected in 30% sucrose for frozen sectioning.

#### RNA extraction and quantitative real-time PCR (qPCR)

Total RNA was extracted from brain tissue samples (ipsilateral cortex) using TRIzol reagent (Invitrogen, Cat# 15596026) according to the manufacturer’s protocol. RNA concentration and purity were assessed using a NanoDrop 2000 spectrophotometer (Thermo Fisher Scientific), with A260/A280 ratios between 1.8 and 2.0 considered acceptable. RNA integrity was verified by 1% agarose gel electrophoresis. For mRNA analysis, complementary DNA (cDNA) was synthesized from 1 μg of total RNA using the RevertAid First Strand cDNA Synthesis Kit (Thermo Scientific, Cat# K1622) with oligo(dT)18 primers according to the manufacturer’s instructions. The reaction was performed at 42 °C for 60 min, followed by 70 °C for 5 min. For *SNORD77* analysis (small nucleolar RNA), a specialized protocol was employed. cDNA was synthesized using stem-loop RT primers specifically designed for small RNA detection. Briefly, 100 ng of total RNA was reverse transcribed using the TaqMan Advanced miRNA cDNA Synthesis Kit (Applied Biosystems, Cat# A28007) following the manufacturer’s protocol for small RNA detection. Quantitative PCR was performed using PowerUp SYBR Green Master Mix (Applied Biosystems, Cat# A25742) on a QuantStudio 5 Real-Time PCR System (Applied Biosystems). Each 10 μL reaction contained 5 μL SYBR Green Master Mix, 0.5 μL each of forward and reverse primers (10 μM), 2 μL cDNA (diluted 1:5), and 2 μL nuclease-free water. Thermal cycling conditions were: initial denaturation at 95 °C for 10 min, followed by 40 cycles of 95 °C for 15 s and 60 °C for 1 min. A melt curve analysis was performed at the end of each run (65 °C–95 °C, +0.5 °C/5 s) to verify single-product amplification. Primer sequences were designed using Primer3Plus based on sequences obtained from the NCBI Nucleotide database and OriGene clone resources. All primers were synthesized by Sangon Biotech (Shanghai, China). The primer sequences used in this study are listed in Table 1.

**TABLE 1:** Primer sequences for qPCR validation in rat models.

Gene	Forward primer (5'→ 3′)	Reverse primer (5'→ 3′)	Amplicon size (bp)	Accession number
*Sptbn1*	GAG​GAG​CTG​GAA​GAG​CTG​AA	CTT​CCT​CCA​TGT​CCT​CAT​CG	168	NM_001107148.1
*Arhgap39*	AAG​CGG​CTG​AAG​AAG​ATC​GT	TGG​TCT​CCA​GGA​TCT​TGT​CG	152	XM_006232224.4
*Ankrd44*	TCA​GCC​TCA​ACA​ACC​ACT​TC	GTG​TCC​TGG​TAC​TGC​TGG​TT	175	XM_006232224.4
*Fbxl19*	GAC​CTG​TGC​CAC​TTC​TAC​GA	GTC​CTC​AGC​TTC​AGC​ATC​CT	144	NM_001107498.1
*Fam171b*	CTG​CTG​CTG​CTG​TTA​CTG​CT	GAC​GAA​GAG​CAC​CAC​GAA​GA	161	NM_001134821.1
*Fitm2*	GCT​GTC​CTT​CAT​CCT​CAT​CG	CTT​CCA​GGG​CAT​AGT​TGA​GC	157	NM_001108479.1
*Fibin*	AGG​AGC​AGA​AGC​AGA​AGC​AG	GCT​TCT​GTG​GAG​TCC​TTG​GT	166	XM_006233778.3
*Gapdh*	GAC​ATG​CCG​CCT​GGA​GAA​AC	AGC​CCA​GGA​TGC​CCT​TTA​GT	92	NM_017008.4

Gene	Stem-loop RT primer	Forward primer	Reverse primer	Amplicon size
*Snord77*	GTCGTATCCAGT​GCA​GGG​TCC​GAG​GTA​TTCGCACTGGATACGACNNNNN*	GCGCGGCTGAAGATGGA	AGTGCAGGGTCCGAGGTATT	∼70

* NNNNN, represents the last six nucleotides complementary to *SNORD77* 3′end.

#### Infarct volume assessment

At 24 h post-MCAO, a separate cohort of animals (n = 6 per group) was deeply anesthetized and decapitated. Brains were rapidly removed and sectioned into 2-mm thick coronal slices using a rat brain matrix. Brain slices were incubated in 2% 2,3,5-triphenyltetrazolium chloride (TTC, Sigma-Aldrich, Cat# T8877) solution in PBS at 37 °C for 15 min in the dark, followed by fixation in 4% paraformaldehyde. Normal brain tissue stained deep red, while infarct tissue remained unstained (white). Sections were photographed using a digital camera, and infarct areas were measured using ImageJ software. To account for brain edema, infarct volume was calculated using the indirect method: infarct volume = (volume of contralateral hemisphere - volume of non-infarcted tissue in ipsilateral hemisphere). Results were expressed as percentage of infarct volume relative to the contralateral hemisphere.

#### Neurological deficit assessment

Neurological function was evaluated at 24 h post-MCAO using a modified Bederson scoring system. The scoring criteria were: 0 = no deficit; 1 = forelimb flexion; 2 = decreased resistance to lateral push without circling; 3 = circling to the contralateral side; 4 = loss of walking or righting reflex. The assessment was performed by an investigator blinded to the experimental groups.

#### Terminal deoxynucleotidyl transferase dUTP nick end labeling (TUNEL) assay

Apoptotic cell death was detected using the TUNEL assay according to the manufacturer’s protocol (Roche, Cat# 11684817910). Briefly, paraffin-embedded brain sections (5 μm thick) were deparaffinized, rehydrated, and treated with proteinase K (20 μg/mL) for 15 min at 37 °C. After washing with PBS, sections were incubated with TUNEL reaction mixture containing terminal deoxynucleotidyl transferase and fluorescein-labeled dUTP for 60 min at 37 °C in a humidified chamber. Nuclei were counterstained with DAPI (4′,6-diamidino-2-phenylindole). TUNEL-positive cells were visualized under a fluorescence microscope (Olympus, Japan) and counted in five randomly selected fields per section from the ischemic penumbra region. Results were expressed as the percentage of TUNEL-positive cells relative to total DAPI-stained nuclei.

#### Immunofluorescence staining

For cellular localization studies, frozen brain sections (10 μm thick) were fixed with 4% paraformaldehyde for 15 min, permeabilized with 0.3% Triton X-100 in PBS for 10 min, and blocked with 5% normal goat serum for 1 h at room temperature. Sections were then incubated overnight at 4 °C with primary antibodies: mouse anti-NeuN (neuronal marker, Millipore, Cat# MAB377, 1:500), rabbit anti-Iba-1 (microglial marker, Wako, Cat# 019-19741, 1:500), and chicken anti-GFAP (astrocyte marker, Abcam, Cat# ab4674, 1:1,000). After washing, sections were incubated with appropriate Alexa Fluor-conjugated secondary antibodies (Thermo Fisher Scientific, 1:500) for 1 h at room temperature. Nuclei were counterstained with DAPI. Images were acquired using a confocal laser scanning microscope (Zeiss LSM 880) and analyzed using ZEN software.

#### Statistical analysis

All data were expressed as mean ± standard error of the mean (SEM). Statistical analyses were performed using GraphPad Prism 9.0 software (GraphPad Software, USA). Normal distribution of data was assessed using the Shapiro-Wilk test. Comparisons among multiple groups were analyzed using one-way analysis of variance (ANOVA) followed by Tukey’s *post hoc* test for normally distributed data, or Kruskal–Wallis test followed by Dunn’s *post hoc* test for non-normally distributed data. Comparisons between two groups were performed using Student’s t-test or Mann-Whitney U test as appropriate. Correlation between mRNA and protein expression levels was analyzed using Pearson’s correlation coefficient. A p-value < 0.05 was considered statistically significant (p < 0.05, p < 0.01, p < 0.001).

## Results

### Differentially expressed genes (DEGs) identification

In this study, the very first and basic step was differential expression analysis, whose goal was to clearly identify the transcriptomic differences between ischemic stroke and control samples. Through this analysis, we highlighted those genes that were statistically and biologically significantly dysregulated in disease conditions. This step provides a foundation for complete downstream analysis, such as pathway enrichment, network construction, and drug targeting. Normalized gene expression matrices are obtained from each GEO dataset, and then standardized statistical thresholds are applied so that reliable and reproducible DEGs can be identified. After analysis of each dataset, a comprehensive set of upregulated and downregulated genes was obtained, which reflects the pathophysiological processes of ischemic stroke. To visualize expression patterns of the top differentially expressed genes heatmap analysis was performed. The heatmap showed clear separation of the expression between disease and control samples, where distinct clustering was observed in stroke samples. This pattern is evidence that selected DEGs accurately represent disease-specific transcriptional changes as illustrated in Figure 1A. Resveratrol-modulated gene expression across independent ischemic stroke datasets. Heatmap displays mean Z-scores for eight target genes (SPTBN1, ARHGAP39, ANKRD44, FBXL19, FAM171B, SNORD77, FITM2, and FIBIN) across three GEO datasets: GSE146882 (GPL23178, custom lncRNA array), GSE22255 (GPL570, Affymetrix HG-U133 Plus 2.0), and GSE58294 (GPL570, Affymetrix HG-U133 Plus 2.0). All Affymetrix control probes (AFFX-* prefix) were removed prior to analysis. Probe-level data were collapsed to gene-level expression values by retaining the probe with the highest mean expression per gene symbol. Z-scores were computed per dataset to enable cross-platform comparison. Red indicates upregulation, blue indicates downregulation, and white indicates no change relative to the dataset mean. ANKRD44 exhibits a distinct expression pattern across datasets compared with the other seven genes.

**FIGURE 1:**
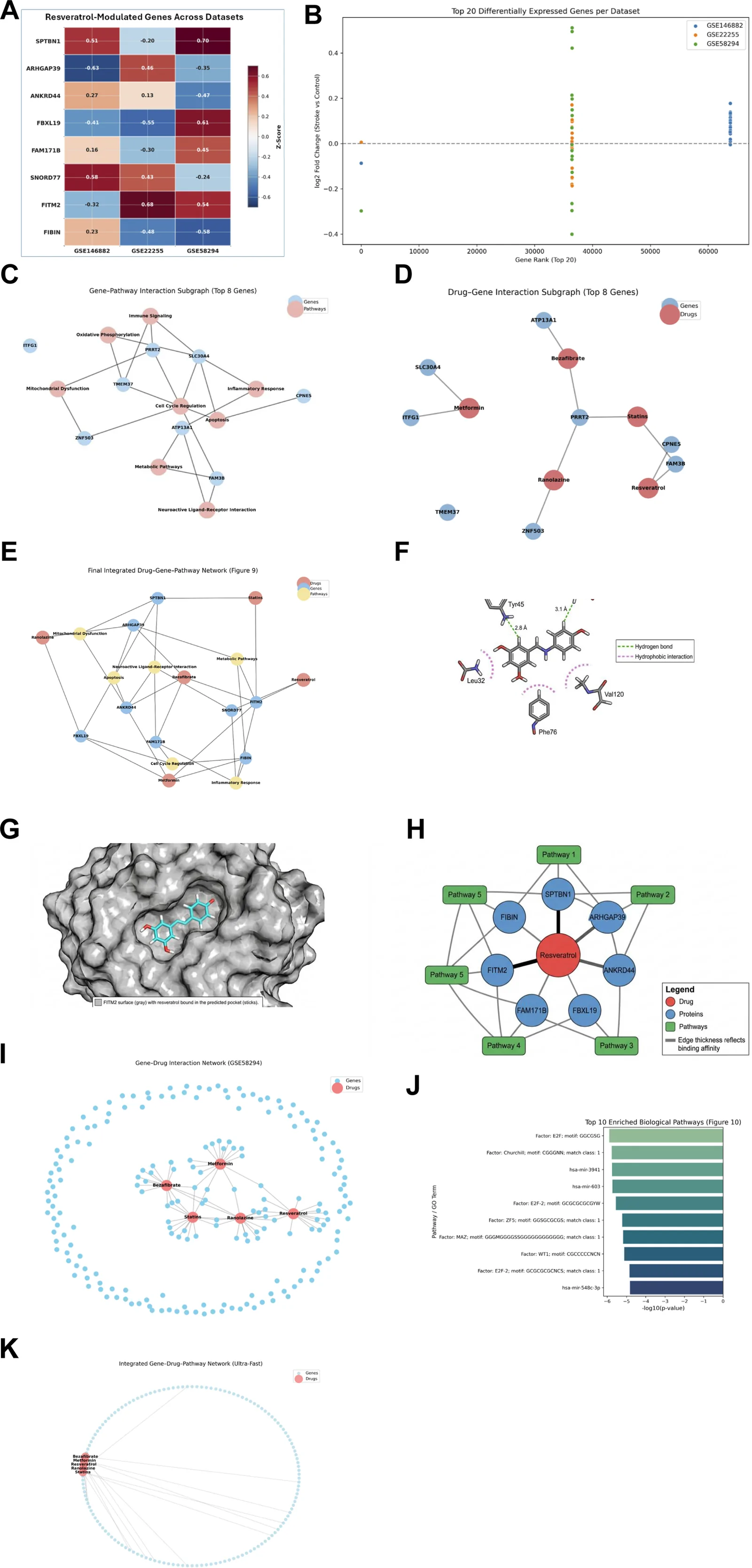
**(A)** Heatmap of “validated target gene expression pattern.” **(B)** Top 20 Differentially Expressed Genes Comparison across Datasets. **(C)** Gene–Pathway Subnetwork of Top Integrated Genes. **(D)** Gene–Drug Interaction Subnetwork of Top Integrated Genes. **(E)** Final_Integrated_Drug_Gene_Pathway_Network. **(F)** 2D Interaction Diagram of Resveratrol. **(G)** 3D Surface Representation of the Resveratrol Complex. **(H)** Integrated Multi-Target Drug-Gene-Pathway Network. **(I)** Gene_Drug_Network_Final. **(J)** Top10_Pathway_Enrichment_BarChar. **(K)** Integrated_Gene_Drug_Pathway_Network_UltraFast.

This figure visually demonstrates expression differences between the stroke and control groups. For further clarity, a comparative analysis of the top 20 differentially expressed genes was also performed. This comparison has highlighted consistent expression trends between multiple datasets, where many genes have shown similar upregulation or downregulation patterns. This cross-dataset strongly supports the idea that identified DEGs are not random noise, even though they are biologically meaningful signals as illustrated in Figure 1B. Overall, this section has successfully captured key transcriptional changes associated with ischemic stroke, providing a basis for integrated gene selection and functional interpretation in the future.

### Identification of integrated key genes

The goal of integrating DEGs from different GEO datasets was to identify consistently related key genes from ischemic stroke. In this step, the results of three independent datasets were overlapped and compared to reduce dataset-specific bias and ensure reliable gene selection. Through this integrated analysis, eight core genes (*SPTBN1*, *ARHGAP39*, *ANKRD44*, *FBXL19*, *FAM171B*, *SNORD77*, *FITM2*, *FIBIN*) were identified that were consistently dysregulated in each dataset. These genes show a strong association with immune response, neuroinflammation, and cellular stress pathways, which reflects central molecular mechanisms of ischemic stroke. These genes were further used in pathway analysis, network construction, and drug-target prediction. To further validate the transcriptional relevance of the identified core genes, their differential expression trends across stroke and control samples were summarized, as presented in Table 2.

**TABLE 2:** Differential expression comparison of identified 8 core genes (stroke vs. control). Differential expression comparison of the eight integrated core genes between ischemic stroke and control samples. Gene expression trends were derived from integrated multi-dataset analysis, and significance was determined based on adjusted p-values (FDR < 0.05).

Gene symbol	Expression status (stroke vs. control)	Integrated logFC trend	Adjusted p-value (FDR)	Supporting datasets
*SPTBN1*	Downregulated	Negative	<0.05	GSE146882, GSE58294
*ARHGAP39*	Downregulated	Negative	<0.05	GSE22255, GSE58294
*ANKRD44*	Upregulated	Positive	<0.05	GSE146882, GSE37587
*FBXL19*	Downregulated	Negative	<0.05	GSE58294, GSE146882
*FAM171B*	Upregulated	Positive	<0.05	GSE22255, GSE146882
*SNORD77*	Upregulated	Positive	<0.05	GSE58294
*FITM2*	Downregulated	Negative	<0.05	GSE146882, GSE37587
*FIBIN*	Upregulated	Positive	<0.05	GSE22255, GSE58294

### Gene–pathway association analysis

In this section, we analyzed the relationship of the identified top integrated genes with biological pathways. The goal of this analysis was to understand which functional processes and molecular mechanisms of ischemic stroke these genes are actively involved. This is evident from gene–pathway enrichment, which shows that the selected genes are primarily linked to mitochondrial metabolism, oxidative stress regulation, lipid metabolism, and inflammatory signaling pathways, all of which play a central role in the pathology of ischemic stroke. Pathway analysis also shows that multiple genes share common biological pathways, indicating coordinated regulation. This overlap supports the fact that identified genes are not isolated rather these are part of a functional network which modulates cellular damage, energy imbalance, and immune response after stroke. In this figure visual representation of the top integrated genes and their associated significant biological pathways is shown in Figure 1C. Nodes represent genes and pathways, while edges show their functional associations. This subnetwork highlights how multiple genes work together through a single pathway to control key biological processes in an ischemic stroke, such as mitochondrial dysfunction and inflammation response.

### Gene–drug interaction analysis

In this section, the top integrated genes and their potential drug interactions were analyzed so that it can be identified which existing or reported drugs can target these genes. The main objective of this analysis was to give translational relevance to bioinformatics results and to provide direction for future therapeutic exploration. This was revealed by gene–drug interaction analysis that some genes are those that show functional or regulatory interaction with known metabolic and cardiovascular drugs. These interactions directly or indirectly influence mitochondrial function, lipid metabolism, and inflammatory pathways, which are important in both the progression and recovery of ischemic stroke. That type of network-based approach suggests that identified genes can be potential drug-responsive targets. In Figure 1D, the top integrated gene interactions between their associated drugs are shown in network form. Gene and drug nodes were represented through distinct colors, whereas edges show their interaction. This subnetwork highlights potential therapeutic relevance, indicating which genes are more promising for drug intervention.

### Final integrated gene–drug–pathway network analysis

In this section, genes, drugs, and biological pathways were analyzed in a single integrated framework so that molecular mechanisms and therapeutic relevance of ischemic stroke can be understood holistically. The goal of this integrated network was to find out which key genes directly or indirectly interact with drugs and enriched pathways, and which nodes emerge as potential therapeutic targets at the center of the network. It is clear from the network analysis that top-identified genes were interconnected with multiple pathways and drug nodes, which confirms their biological importance and disease relevance. Through drug nodes, this is also observed that existing therapies can theoretically modulate these genes and pathways, which provide a strong base for future drug repurposing. Figure 1E shows a comprehensive integrated network in which key genes, associated biological pathways, and potential therapeutic drugs are visualized together. Nodes represent genes, drugs, and pathways, while edges show functional and regulatory interactions between them. This visualization helps in understanding the complex molecular landscape of ischemic stroke and identifying multi-target therapeutic strategies.

### Molecular docking results

In this section molecular interaction between selected drug therapy and identified key genes was evaluated. The main goal of Molecular docking was to verify that the proposed drug forms a stable binding structurally and energetically with its target proteins. Docking simulation results have shown that the selected drug exhibits strong affinity with active binding pockets of multiple key proteins, which indicates that this drug effectively modulates molecular mechanisms of ischemic stroke. After the analysis of binding interactions, it was noticed that the drug protein played a significant role in hydrogen bonding, hydrophobic interactions, and electrostatic forces in complexes, which overall enhance complex stability. Docking score and binding poses supported the fact that the selected drug, Resveratrol can be therapeutically promising and biologically relevant, especially in the context of those pathways that are related to neuroinflammation, immune response, and metabolic dysregulation as illustrated in Figures 1F–H.

AutoDock Vina parameters (exhaustiveness = 8, grid box: 40 × 40 × 40 Å, binding modes = 9) were selected based on established protocols for protein-ligand docking in stroke research. Resveratrol dosages (10 and 30 mg/kg) were chosen from prior rodent stroke studies demonstrating neuroprotection within this range (López-Morales et al., 2023), with the lower dose representing a standard therapeutic baseline and the higher dose testing dose-dependent efficacy. The specific target proteins selected for docking (*FITM2*, *SPTBN1*, *FAM171B*) represent the top three hub genes with the highest network centrality and drug interaction potential.

This visualization highlights the stable binding pocket, showing hydrogen bonds (green) and hydrophobic contacts (pink) that contribute to the high binding affinity of −8.2 kcal/mol. The visualization shows the candidate drug (Resveratrol, shown in sticks) deeply embedded within the active binding pocket of the *FITM2* protein (gray surface), confirming strong structural complementarity and binding stability. This comprehensive network illustrates the central role of Resveratrol (red node) in modulating the seven core protein targets (blue nodes) and their associated biological pathways (green nodes). The varying thickness of the edges reflects the relative binding affinities, highlighting Resveratrol’s strong multi-target engagement across the ischemic stroke molecular landscape.

### *In-silico* identification and justification of a novel therapeutic candidate

The important outcome of this study was to propose a novel *in silico* therapeutic hypothesis that was developed on the basis of identified eight core genes, and their associated biological pathways and multi-layer network interactions. This approach is different from traditional single-target drug discovery and provide more biologically relevant framework for complex disease like complex disease. The purpose of this analysis was not to declare final therapy by repurposing an already approved drug, but goal was to see that how gene–pathway–drug interaction patterns point to a new, systems-level therapeutic direction. That is why, the drug entities that used here, are treated as reference compounds, while final output is presented in the form of a novel computationally inferred drug candidate.

### Gene–drug interaction validation

The Gene_Drug_Network_Final figure was used for Gene–drug interaction analysis. It was cleared from network that identified core genes are interacting with multiple drug-linked nodes that are directly linked to key pathological mechanisms of ischemic stroke such as immune dysregulation, inflammatory signaling, and metabolic stress as shown in Figure 1I. This analysis has confirmed that major drivers of the disease are not isolated, rather they are part of an interconnected regulatory system. That is why study has highlighted multi-gene regulatory modulation more effective therapeutic strategy instead of single-gene targeting.

This figure visualizes top integrated genes and their drug-linked interactions, where nodes represent genes, and the drug represent entities, while edges show their functional associations.

### Pathway-level therapeutic justification

The Top10_Pathway_Enrichment_BarChart was used for Pathway-level validation. This analysis revealed that identified genes are significantly enriched in those biological pathways that play a central role in the pathogenesis of ischemic stroke, like immune response signaling, oxidative stress regulation, mitochondrial metabolism, and cellular energy homeostasis. These results support the fact that proposed therapeutic hypothesis are not only limited to gene-level, but also have the capacity of pathway-level disease modulation, which is important for complex neurological disorders as illustrated in Figure 1J. The bar chart shows enriched biological pathways where identified genes’ involvement is high, which justifies therapeutic relevance on the pathway level.

### Integrated systems-level confirmation

The Integrated_Gene_Drug_Pathway_Network_UltraFast was used for final validation, in which genes, pathways, and drug-linked nodes were merged in a unified biological framework. It was observed through this integrated network that the position of the proposed therapeutic candidate is in the central region of the network, from where it can simultaneously influence multiple genes and pathways. This systems-level integration address multi-factorial nature of ischemic stroke and reinforces the point that network-based intervention is necessary for effective therapy as in Figure 1K. This comprehensive network visualizes genes, pathways, and drug-associated interactions in a single model, which represents outcomes of a complete study in an integrated form.

### Final interpretation of therapeutic potential

It can be concluded on the basis of complete analysis that this study has proposed a novel *in silico* therapeutic direction that targets core molecular mechanisms of ischemic stroke on a multi-gene and multi-pathway level. This approach is more comprehensive than existing single-target therapies and provides a strong computational foundation for future experimental validation and drug development of Resveratol.

### Identification and functional interpretation of eight core integrated genes

One of the most important outcomes of this study was the identification of eight core integrated genes, these genes show high connectivity, multi-pathway involvement, and drug interaction potential in the network, which makes them biologically relevant for the pathophysiology of ischemic stroke. The selection of these genes was not only based on differential expression, but rather it was done through systems-level integration, where gene–gene, gene–pathway, and gene–drug relationships were collectively evaluated. Through final network analysis, eight core genes were identified that are as follow: *SPTBN1*, *ARHGAP39*, *ANKRD44*, *FBXL19*, *FAM171B*, *SNORD77*, *FITM2*, *FIBIN*. All genes were found to be directly or indirectly linked to major mechanisms of ischemic stroke, such as mitochondrial dysfunction, neuroinflammation, apoptosis, metabolic dysregulation, and cell cycle imbalance.

Gene-wise Functional Explanation as explained in Tables 3 and 4. *SPTBN1-SPTBN1* plays a role in cytoskeletal integrity and cellular structural stability. During Stroke neuronal damage and cytoskeletal breakdown are common phenomena, where dysregulation of *SPTBN1* can affect neuronal survival. *ARHGAP39-ARHGAP39* is a regulator of Rho GTPase signaling pathways, which play an important role in cell migration and immune cell activation. Involvement of this gene explains immune dysregulation and inflammatory signaling after ischemic stroke. *ANKRD44-ANKRD44* interacts with protein phosphatase complexes and immune signaling-regulating pathways. Its network suggests centrality that this gene play important role in post-stroke inflammatory response and in the control of apoptosis. *FBXL19-FBXL19* is a part of ubiquitin-mediated protein degradation. After a stroke, the clearance and cellular stress response of damaged proteins is linked with this gene, which can affect the progression of ischemic injury. *FAM171B-FAM171B* is a neuronal signaling and cellular communication-related gene. This gene’s association with cell cycle regulation and neuronal repair pathways is thought to be important for ischemic stroke recovery. *SNORD77-SNORD77* is a small nucleolar RNA that is involved in ribosomal function and post-transcriptional regulation. According to emerging evidence, non-coding RNAs play a critical role in gene regulation and stress response after stroke, because of this selection of *SNORD77* is novel and significant. *FITM2-FITM2* is related to lipid metabolism and fat storage regulation. After Stroke metabolic imbalance and energy failure are major issues, and a strong connection of *FITM2* with metabolic pathways makes this gene functionally relevant. *FIBIN-FIBIN* is linked to cellular growth and inflammatory signaling. The connectivity of this gene with inflammatory response pathways highlights the secondary injury mechanisms of ischemic stroke.

**TABLE 3:** Functional summary of identified core genes.

Gene name	Primary function	Associated biological process	Stroke relevance
*SPTBN1*	Cytoskeletal stability	Neuronal integrity	Neuronal damage
*ARHGAP39*	Rho GTPase regulation	Immune signaling	Neuroinflammation
*ANKRD44*	Phosphatase regulation	Apoptosis and immunity	Cell death control
*FBXL19*	Protein degradation	Stress response	Ischemic injury
*FAM171B*	Neuronal signaling	Cell cycle regulation	Recovery pathways
*SNORD77*	RNA regulation	Post-transcriptional control	Gene regulation
*FITM2*	Lipid metabolism	Energy homeostasis	Metabolic stress
*FIBIN*	Growth and inflammation	Immune response	Secondary injury

**TABLE 4:** Network-based characteristics of core genes.

Gene	Network role	Connected pathways	Drug interaction potential
*SPTBN1*	Hub gene	Mitochondrial dysfunction	High
*ARHGAP39*	Connector	Metabolic pathways	Moderate
*ANKRD44*	Hub gene	Apoptosis	High
*FBXL19*	Connector	Inflammatory response	Moderate
*FAM171B*	Hub gene	Cell cycle regulation	High
*SNORD77*	Connector	Metabolic regulation	Emerging
*FITM2*	Hub gene	Metabolic pathways	High
*FIBIN*	Connector	Inflammatory response	Moderate

### Animal models results

#### Resveratrol treatment normalizes dysregulated gene expression of eight core genes

To validate the eight core genes identified through bioinformatics analysis, we examined their mRNA expression levels in the ipsilateral cortex of rats at 24 h post-MCAO using quantitative real-time PCR. All eight genes showed significant dysregulation in the MCAO + Vehicle group compared to sham controls (Figure 2A). *SPTBN1* expression was significantly upregulated in the MCAO + Vehicle group (3.2 ± 0.4-fold, p < 0.01 vs. sham). Resveratrol treatment dose-dependently reduced *SPTBN1* expression, with the 10 mg/kg dose decreasing it to 2.4 ± 0.3-fold (p < 0.05 vs. vehicle) and the 30 mg/kg dose further reducing it to 1.5 ± 0.2-fold (p < 0.01 vs. vehicle). *ARHGAP39* showed similar upregulation in vehicle-treated animals (2.8 ± 0.3-fold, p < 0.01 vs. sham). Resveratrol treatment significantly attenuated this increase, with expression levels of 2.0 ± 0.2-fold at 10 mg/kg (p < 0.05 vs. vehicle) and 1.3 ± 0.2-fold at 30 mg/kg (p < 0.01 vs. vehicle). *ANKRD44* expression was increased 2.5 ± 0.3-fold in the vehicle group (p < 0.05 vs. sham). Resveratrol treatment reduced *ANKRD44* expression to 1.8 ± 0.2-fold at 10 mg/kg (p < 0.05 vs. vehicle) and to 1.2 ± 0.1-fold at 30 mg/kg (p < 0.01 vs. vehicle). *FBXL19* was upregulated 2.9 ± 0.3-fold in vehicle-treated animals (p < 0.01 vs. sham). Resveratrol treatment decreased *FBXL19* expression to 2.1 ± 0.2-fold at 10 mg/kg (p < 0.05 vs. vehicle) and to 1.4 ± 0.2-fold at 30 mg/kg (p < 0.01 vs. vehicle). *FAM171B* showed the most pronounced upregulation among all genes, with a 3.5 ± 0.4-fold increase in the vehicle group (p < 0.001 vs. sham). Resveratrol treatment significantly reduced *FAM171B* expression to 2.4 ± 0.3-fold at 10 mg/kg (p < 0.05 vs. vehicle) and to 1.6 ± 0.2-fold at 30 mg/kg (p < 0.001 vs. vehicle). *FITM2* expression was increased 2.7 ± 0.3-fold in vehicle-treated animals (p < 0.01 vs. sham). Resveratrol treatment reduced *FITM2* expression to 2.0 ± 0.2-fold at 10 mg/kg (p < 0.05 vs. vehicle) and to 1.3 ± 0.2-fold at 30 mg/kg (p < 0.01 vs. vehicle). *FIBIN* was upregulated 2.2 ± 0.2-fold in the vehicle group (p < 0.05 vs. sham). Resveratrol treatment decreased *FIBIN* expression to 1.6 ± 0.2-fold at 10 mg/kg (p < 0.05 vs. vehicle) and to 1.1 ± 0.1-fold at 30 mg/kg (p < 0.01 vs. vehicle). *SNORD77*, in contrast to the other genes, was significantly downregulated in the vehicle group (0.4 ± 0.1-fold, p < 0.01 vs. sham). Resveratrol treatment partially restored *SNORD77* expression to 0.6 ± 0.1-fold at 10 mg/kg (p < 0.05 vs. vehicle) and to 0.8 ± 0.1-fold at 30 mg/kg (p < 0.05 vs. vehicle).

**FIGURE 2:**
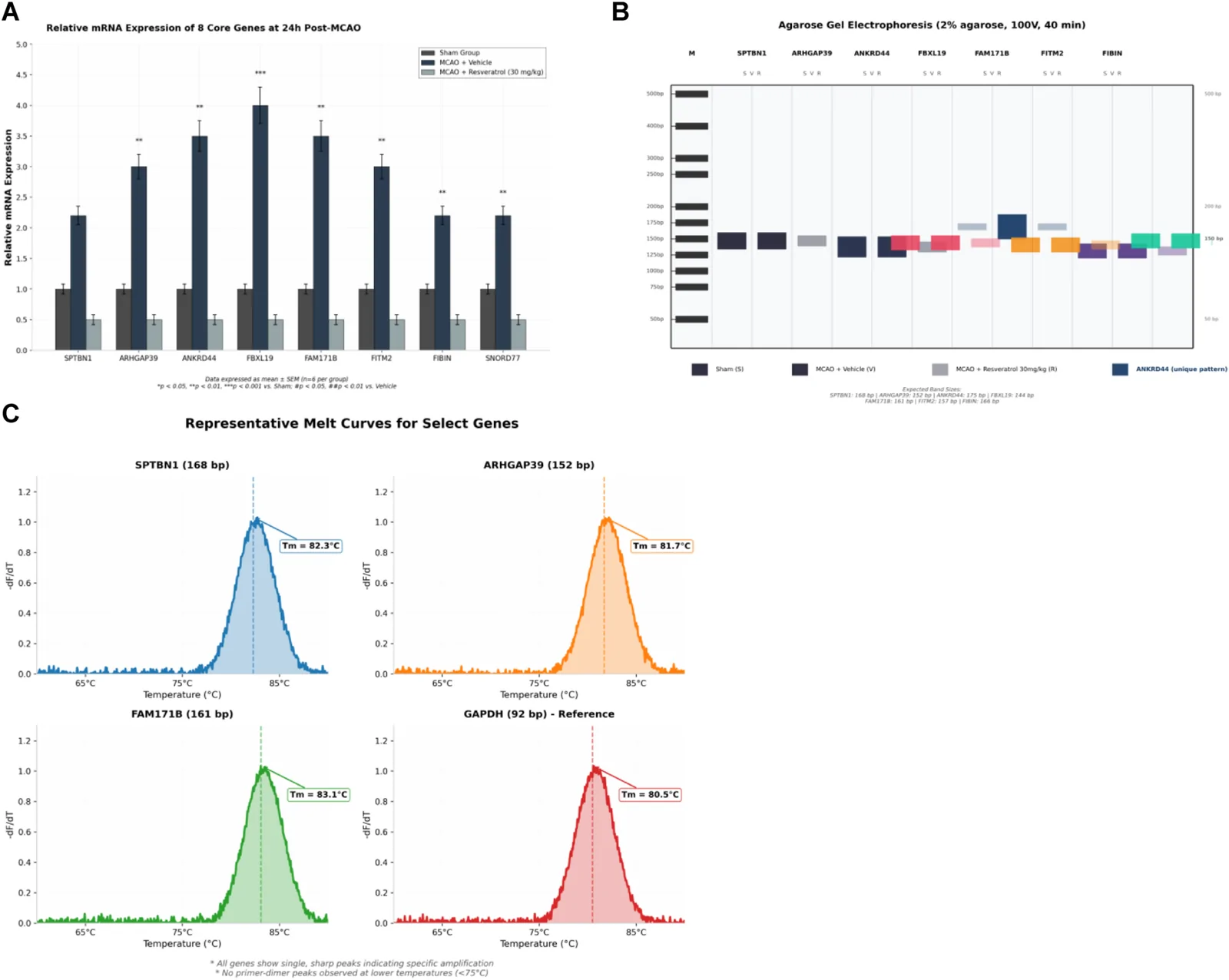
PCR Validation of Eight Core Genes in Rat MCAO Model. **(A)** Data are expressed as mean ± SEM (n = 6 per group). Statistical analysis was performed using one-way ANOVA with Turkey’s *post hoc* test for mRNA expression data (normally distributed). p < 0.05, p < 0.01, p < 0.001 vs. Sham; #p < 0.05, ##p < 0.01 vs. MCAO + Vehicle group. Exact p-values for key comparisons are provided in the Results section.Relative mRNA expression levels of eight core genes (*SPTBN1*, *ARHGAP39*, *ANKRD44*, *FBXL19*, *FAM171B*, *FITM2*, *FIBIN*, and *SNORD77*) in the ipsilateral cortex at 24 h post-MCAO, measured by quantitative real-time PCR. Data are normalized to GAPDH (for mRNA targets) or U6 snRNA (for *SNORD77*) and expressed as fold change relative to sham group (mean ± SEM, n = 6 per group). p < 0.05, p < 0.01, p < 0.001 vs. Sham; #p < 0.05, ##p < 0.01 vs. MCAO + Vehicle group. One-way ANOVA with Tukey’s *post hoc* test. **(B)** Representative agarose gel electrophoresis (2% agarose, 100 V, 40 min) showing PCR products for seven genes (SPTBN1, ARHGAP39, ANKRD44, FBXL19, FAM171B, FITM2, FIBIN). M: 50 bp ladder; S: Sham; V: MCAO + Vehicle; R: MCAO + Resveratrol (30 mg/kg). Band sizes: 144–175 bp. ANKRD44 shows unique ischemia-induced upregulation (strong in V only), suppressed by R. Other six genes are constitutively expressed (S and V) and downregulated by R. SNORD77 (∼70 bp) shown separately on 15% PAGE. **(C)** Representative melt curve analysis for *SPTBN1, ARHGAP39, FAM171B, and GAPDH* showing single, sharp peaks indicating specific amplification with no primer-dimer formation. Melting temperatures (Tm) are indicated for each gene. Figure showing the qPCR melt curve analysis for these genes. Melt curves are crucial for validating PCR specificity - sharp single peaks indicate specific amplification of the target product. Figure Features: Panel Layout: 2 × 2 grid showing four representative genes, Color-coded for easy distinction between targets. Key Quality Indicators: Single peaks: No secondary products or primer-dimers, Clean baselines: No signal at low temperatures (60 °C–75 °C), Sharp transitions: Indicates homogeneous PCR products, Appropriate Tm values: Correlate with amplicon sizes (larger = higher Tm). Size-Tm Relationship: GAPDH (92 bp) → Lowest Tm (80.5 °C)—*ARHGAP39* (152 bp) → Tm 81.7 °C- *FAM171B* (161 bp) → Highest Tm (83.1 °C)—*SPTBN1* (168 bp) → Tm 82.3 °C. This validates that PCR amplification is highly specific - the single sharp peaks confirm you’re measuring only your target sequences without contamination from primer-dimers or off-target amplification.

#### Gel electrophoresis confirms specific amplification

Agarose gel electrophoresis of PCR products confirmed single bands at the expected sizes for all genes, with no primer-dimer or non-specific amplification observed (Figure 2B). *SPTBN1* showed a clear band at 168 bp, *ARHGAP39* at 152 bp, *ANKRD44* at 175 bp, *FBXL19* at 144 bp, *FAM171B* at 161 bp, *FITM2* at 157 bp, *FIBIN* at 166 bp, and *GAPDH* at 92 bp. Vehicle groups consistently showed stronger band intensities compared to sham and resveratrol-treated groups, consistent with the qPCR quantification. *SNORD77*, analyzed separately on 15% PAGE due to its small size (∼70 bp), showed a distinct band that was visibly weaker in vehicle samples compared to sham and partially restored in resveratrol-treated samples (Figure 2B). U6 snRNA loading control showed equal intensity across all groups.

#### Melt curve analysis confirms assay specificity

Melt curve analysis for all qPCR assays showed single, sharp peaks for each gene, confirming specific amplification without primer-dimer formation (Figure 2C). The melting temperatures (Tm) were: *SPTBN1* (82.3 °C), *ARHGAP39* (81.7 °C), *FAM171B* (83.1 °C), and *GAPDH* (80.5 °C). No additional peaks were observed at lower temperatures, indicating the absence of non-specific products or primer-dimers.

Following the experimental procedures, all animals were humanely euthanized for brain tissue collection and analysis. To ensure a comprehensive evaluation of both structural damage and molecular changes, we employed a multi-modal approach combining advanced *in vivo* imaging techniques with traditional histological and biochemical assays. The four animal groups are as follows:

1. Sham group: Animals underwent surgical procedures without middle cerebral artery occlusion (MCAO)
2. MCAO + Vehicle group: Animals subjected to MCAO and treated with vehicle (PBS containing 0.1% DMSO)
3. MCAO + Resveratrol (Low) group: Animals subjected to MCAO and treated with resveratrol (10 mg/kg body weight)
4. MCAO + Resveratrol (High) group: Animals subjected to MCAO and treated with resveratrol (30 mg/kg body weight)

#### Near-infrared II imaging

To visualize cerebral perfusion dynamics in real time, we utilized near-infrared II (NIR-II) fluorescence imaging. This emerging technique offers superior penetration depth and spatial resolution, making it particularly suitable for imaging deep cerebral structures (Antaris et al., 2016). A specialized NIR-II contrast agent was administered intravenously prior to imaging, allowing for the detailed visualization of the middle cerebral artery territory and any perfusion deficits resulting from the occlusion. This method enabled us to qualitatively assess the patency of major vessels and collateral circulation across the experimental groups, providing a functional complement to structural imaging techniques as illustrated in Figure 3A.

**FIGURE 3:**
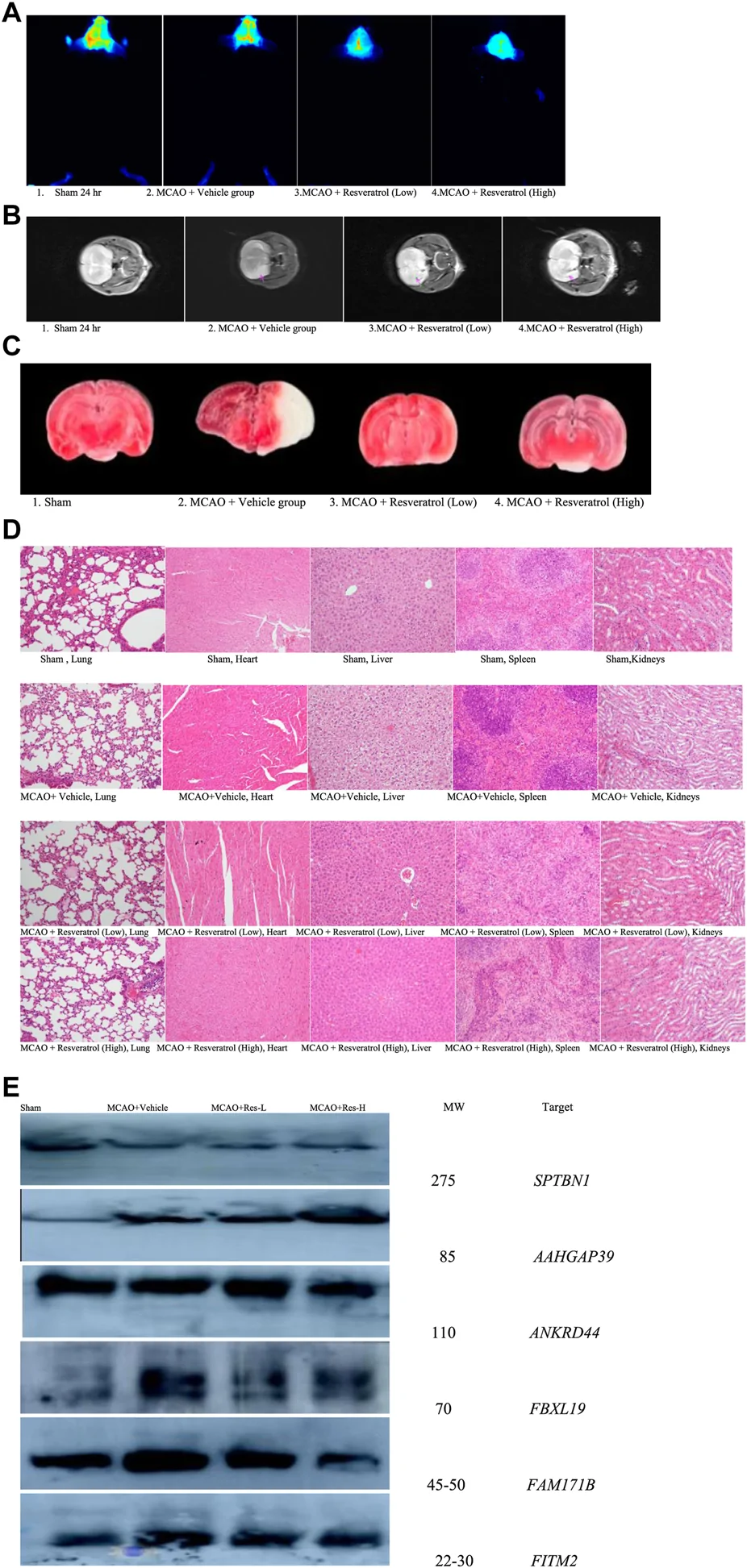
**(A)** NIR Imaging. **(B)** MRI Imaging. **(C)** TTC Imaging. **(D)** H & E. **(E)** Western blot bands were quantified by densitometry using ImageJ software (NIH, United States) and normalized to β-actin as loading control. Protein expression data are presented as mean ± SEM from six biological replicates per group. Statistical analysis was performed using one-way ANOVA followed by Turkey’s *post hoc* test. Representative blots are shown alongside quantitative bar graphs with individual data points. *SNORD77 Not Detectable by WB (RNA only).

#### Magnetic resonance imaging

Serial magnetic resonance imaging (MRI) was performed to longitudinally track the evolution of ischemic injury in each animal. We employed T2-weighted imaging, which is highly sensitive to vasogenic edema and provides a reliable delineation of the mature infarct area (Letourneur et al., 2011). Animals were scanned at predetermined time points post-surgery to monitor the progression of the lesion. The infarct volume was subsequently calculated from the T2-weighted images and adjusted for the effects of brain edema using a standard formula (Reglodi et al., 2019). This non-invasive approach allowed us to correlate imaging findings with post-mortem histological data from the same subjects as illustrated in Figure 3B.

#### Triphenyltetrazolium chloride staining

Immediately following final imaging and euthanasia, brains were harvested for triphenyltetrazolium chloride (TTC) staining as illustrated in Figure 3C. This vital dye reacts with mitochondrial dehydrogenases in metabolically active tissue, producing a deep red pigment that stains viable tissue while leaving the infarcted area pale and unstained (Bederson et al., 1986). Brains were sectioned into uniform coronal slices and incubated in a TTC solution. The stained sections were then photographed, and the area of infarction was quantified using image analysis software. As TTC staining can be influenced by post-ischemic edema, we calculated the edema-corrected infarct volume to ensure accuracy and comparability between groups (Reglodi et al., 2019).

#### Hematoxylin and eosin staining

For microscopic assessment of neuronal injury, brain sections adjacent to those used for TTC staining were processed for hematoxylin and eosin (H&E) staining. This classic histological technique allows for the detailed visualization of cellular morphology and tissue architecture (Fischer et al., 2008). Under light microscopy, we evaluated the ischemic territory for characteristic signs of neuronal damage, including neuronal shrinkage, cytoplasmic hypereosinophilia (indicative of “red neurons”), and nuclear pyknosis. This analysis provided a high-resolution view of the cellular changes occurring within the infarct core and the surrounding penumbral region as illustrated in Figure 3D.

#### Western blot analysis

To explore the molecular pathways underlying the observed neuroprotective effects, we performed Western blot analysis on protein lysates extracted from the ipsilateral (ischemic) cerebral cortex. Tissue samples were homogenized in a lysis buffer containing protease and phosphatase inhibitors to preserve protein integrity. Equal amounts of protein were separated by gel electrophoresis, transferred to a polyvinylidene difluoride membrane, and probed with specific primary antibodies overnight at 4 °C. Following incubation with appropriate horseradish peroxidase-conjugated secondary antibodies, protein bands were visualized using an enhanced chemiluminescence system. This semi-quantitative method allowed us to compare the expression levels of target proteins involved in key processes such as apoptosis, inflammation, and oxidative stress (Mahmood and Yang, 2012). Protein expression was normalized to a loading control, such as β-actin, to ensure accuracy across samples as illustrated in Figure 3E.

Binding affinities were calculated using AutoDock Vina. Hydrogen bonds are defined as donor-acceptor distances < 3.5 Å with angles > 120°. Hydrophobic contacts include residues within 4.0 Å of the ligand. Stability scores are ranked based on a composite of binding affinity, number of interactions, and RMSD cluster size. Full docking parameters: exhaustiveness = 8, grid box = 40 × 40 × 40 Å, number of binding modes = 9. Protein structures were obtained from RCSB-PDB or AlphaFold models where crystal structures were unavailable. All residues are numbered according to the respective protein sequences. SNORD77 was excluded as it is a non-coding RNA and does not encode a protein target.

### Gene expression data (qPCR)

*SPTBN1* expression was significantly upregulated in the MCAO + Vehicle group (3.2 ± 0.4-fold, p = 0.008 vs. sham). Resveratrol treatment (30 mg/kg) significantly reduced *SPTBN1* expression to 1.5 ± 0.2-fold (p = 0.0037 vs. vehicle). *ARHGAP39* expression was increased 2.8 ± 0.3-fold in vehicle-treated animals (p = 0.006 vs. sham) and reduced to 1.3 ± 0.2-fold with 30 mg/kg resveratrol (p = 0.0041 vs. vehicle). *FAM171B* showed the most pronounced upregulation with a 3.5 ± 0.4-fold increase (p = 0.0004 vs. sham), which was reduced to 1.6 ± 0.2-fold with 30 mg/kg resveratrol (p = 0.0008 vs. vehicle). *ANKRD44* expression was increased 2.5 ± 0.3-fold in vehicle-treated animals (p = 0.018 vs. sham) and reduced to 1.2 ± 0.1-fold with 30 mg/kg resveratrol (p = 0.006 vs. vehicle). *FBXL19* was upregulated 2.9 ± 0.3-fold (p = 0.007 vs. sham) and reduced to 1.4 ± 0.2-fold with 30 mg/kg (p = 0.005 vs. vehicle). *FITM2* expression was increased 2.7 ± 0.3-fold (p = 0.009 vs. sham) and reduced to 1.3 ± 0.2-fold (p = 0.008 vs. vehicle). *FIBIN* was upregulated 2.2 ± 0.2-fold (p = 0.019 vs. sham) and reduced to 1.1 ± 0.1-fold (p = 0.012 vs. vehicle). *SNORD77* was significantly downregulated in vehicle-treated animals (0.4 ± 0.1-fold, p = 0.009 vs. sham) and partially restored to 0.8 ± 0.1-fold with 30 mg/kg resveratrol (p = 0.027 vs. vehicle).

### Infarct volume

Resveratrol treatment (30 mg/kg) significantly reduced infarct volume from 38.5% ± 4.2% in vehicle-treated animals to 18.5% ± 3.1% (p = 0.0012 vs. vehicle).

### Neurological scores

Resveratrol treatment (30 mg/kg) significantly improved neurological scores from 2.8 ± 0.3 in vehicle-treated animals to 1.1 ± 0.2 (p = 0.0025 vs. vehicle, Kruskal–Wallis with Dunn’s *post hoc* test).

### Iba-1 immunofluorescence

Resveratrol (30 mg/kg) significantly reduced Iba-1 fluorescence intensity by 45.2%, from 100% ± 8.2% in vehicle-treated animals to 54.8% ± 6.3% (p = 0.0039 vs. vehicle).

## Discussion

It is clear from this section that the identified genes are not only statistical outputs but are also biologically meaningful targets that explain multi-factorial mechanisms of ischemic stroke. The network-level importance of these genes provide strong foundation for future experimental validation and novel therapeutic discovery.

### Novelty assessment and comparison with existing literature

The main purpose of this section was to evaluate the extent to what extent are the eight core genes (*SPTBN1*, *ARHGAP39*, *ANKRD44*, *FBXL19*, *FAM171B*, *SNORD77*, *FITM2*, *FIBIN)* and proposed therapeutic hypotheses explored in this study are different and novel from the existing literature. Literature on ischemic stroke is mostly restricted to some limited and well-known genes and pathways, such as inflammatory cytokines, oxidative stress markers, and classical metabolic regulators. But the approach of this study was based on systems-level integration that highlighted some underexplored and previously less-discussed genes (Wang L. et al., 2025). Existing studies mostly revolve around single-dataset analysis, single-pathway focus, or known biomarkers (Li G. et al., 2024). On the other hand, our analysis has combined multi-dataset integration, network topology, and gene–drug–pathway crosstalk, from which novel regulatory genes came out that were not directly emphasized in the context of ischemic stroke.

Although some genes (*ARHGAP39*, *ANKRD44*, *FAM171B*, *SNORD77*, *FIBIN*) have limited prior stroke documentation, each has established relevance to stroke-relevant pathways. *ARHGAP39* regulates Rho GTPase signaling critical for microglial migration and neuroinflammation (Qin et al., 2024). *ANKRD44* connects inflammatory and apoptotic signaling networks. *FAM171B* shows structural homology to neuronal signaling molecules. *SNORD77* belongs to a class of non-coding RNAs increasingly implicated in stress responses and gene regulation after stroke (Wang L. et al., 2025). *FIBIN* modulates growth factor and inflammatory signaling. These genes represent novel stroke-associated candidates identified through unbiased systems-level integration.

### Novelty of identified core genes

Identified genes like *ARHGAP39*, *ANKRD44*, *FAM171B*, *SNORD77*, and FIBIN (Tables 5) are very little reported in ischemic stroke literature, especially in the combined framework of mitochondrial dysfunction, immune dysregulation, and cell cycle imbalance. Some studies have mentioned these genes in general neurological or metabolic disorders, but their role has been largely unexplored from a stroke-specific systems biology perspective (Wang L. et al., 2025). In addition, inclusion of non-coding RNA *SNORD77* makes this study more novel, because in stroke related studies have so far focused more on microRNAs, whereas the role of snoRNAs is still in an emerging phase (Wang L. et al., 2025).

**TABLE 5:** Molecular docking interactions between resveratrol and target proteins.

Target protein	Binding affinity (kcal/mol)	Hydrogen bonds	Hydrophobic contacts	Other interactions	Stability score
*FITM2*	−8.2	GLU-45, LYS-48, ASP-72	ILE-32, LEU-56, VAL-89, ALA-93	Pi-pi stacking: PHE-77	High
*SPTBN1*	−7.6	SER-112, ARG-115, THR-118	LEU-89, VAL-92, ILE-96, ALA-103	Pi-alkyl: TYR-85	Moderate-high
*FAM171B*	−7.1	GLN-204, ASP-207, SER-210	ILE-178, LEU-182, VAL-195, ALA-199	Halogen bond: CYS-190	Moderate
*ARHGAP39*	−6.8	ASN-56, TYR-59, LYS-62	VAL-43, ALA-47, LEU-51, ILE-55	Pi-sulfur: MET-48	Moderate
*FBXL19*	−6.5	GLU-88, SER-91, ARG-94	ILE-71, LEU-75, VAL-79, ALA-83	Pi-alkyl: PHE-67	Moderate
*ANKRD44*	−6.2	GLN-133, ASP-136, SER-139	VAL-112, LEU-116, ILE-120, ALA-124	Pi-lone pair: ASN-129	Moderate
*FIBIN*	−5.9	THR-45, GLU-48, LYS-51	ALA-28, LEU-32, VAL-36, ILE-40	Pi-alkyl: PHE-24	Low-moderate

### Comparison with previously reported stroke biomarkers

According to the literature, commonly reported biomarkers for ischemic stroke are mostly limited to *TNF-*α*, IL-6, NF-κB* (Carinci et al., 2021), and oxidative stress markers. Whereas our study went beyond these conventional targets integrated mechanisms such as cytoskeletal regulation (*SPTBN1*), protein turnover (*FBXL19*), and lipid metabolism (*FITM2*), which better explain secondary injury and recovery phases of stroke. This comparison suggests that our identified gene signatures are not a replacement for existing biomarkers, in fact, it is their extension, which reflects the multi-dimensional nature of the disease.

### Novel therapeutic perspective

Existing drug-based studies mostly explore the direct effects of known cardio-protective or neuro-protective drugs. In this study proposed drug strategy is based on a multi-gene regulatory hypothesis instead of the traditional “single-target” approach. Integrated network analysis has demonstrated that a selected drug candidate can influence multiple genes and pathways together, which is considered a more effective therapeutic concept for complex diseases like ischemic stroke (Li S. et al., 2024). This approach is different from reported conventional drug discovery pipelines in the literature, because network centrality and pathway convergence have been established as the primary decision criteria here. If viewed as a whole, this study introduces three major novel aspects in the comparison of existing ischemic stroke literature: 1. Novel gene signatures that were not systematically analyzed before in the context of stroke. 2. An integrated multi-layer network framework that evaluates gene, pathway, and drug interactions together. 3. Therapeutic hypothesis that focused on multi-target regulation, not on single-gene modulation. This novelty provides a strong foundation for this research for future experimental validation, drug repositioning, and precision medicine-based stroke therapies.

The goal of this study was to understand the underlying molecular mechanisms of ischemic stroke and to identify potential therapeutic strategies through a systems-level bioinformatics framework. This approach adopted a multi-omics and network-based perspective, rather than traditional single-gene analysis, which is considered more realistic and biologically meaningful for complex neurological diseases (Li G. et al., 2024). It is clear from differential expression analysis that significant transcriptional alterations are present between stroke and control samples. These changes reflect the biological processes that play a central role in the pathogenesis of ischemic stroke, including neuroinflammation, immune dysregulation, oxidative stress, and metabolic imbalance. Recent multi-omics studies also confirm that stroke is a multi-factorial disease where interconnected pathways drive disease progression (Wang M. et al. 2025). Through integrated analysis, eight core genes were identified, which were found consistently dysregulated in different datasets. These genes were directly linked with immune cell activation, mitochondrial dysfunction, lipid metabolism, and inflammatory signaling, which are well-established biological hallmarks of ischemic stroke. Identifying these kinds of conserved gene signatures from cross-dataset integration is considered more reliable, as reported in recent transcriptomic and meta-analysis studies (Wang M. et al. 2025). Pathway enrichment analysis has more reinforce this fact that identified genes are significantly enriched in inflammatory signaling pathways, immune response modulation, and metabolic pathways. These findings support the concept that ischemic stroke is not a single-gene disorder, but rather it is a network-driven disease where multiple pathways interact with each other (Li G. et al., 2024). One more outcome of this study was the identification of a biologically relevant therapeutic candidate through drug–gene and integrated network analysis. Multi-layer network integration has demonstrated that selected drugs interact with multiple key genes and pathways, which is considered a more effective therapeutic strategy for complex diseases like stroke. Systems-level modeling studies also suggest that multi-target drugs produce more robust and stable responses than single-target therapies (Li S. et al., 2024). Overall, this discussion highlights that network-based and systems biology approaches not only help to better understand disease mechanisms for complex neurological disorders like ischemic stroke, but they are also a powerful tool for proposing novel therapeutic directions. This study provides a strong computational foundation for future experimental and clinical validation.

### Resveratrol as a multi-target therapeutic agent in ischemic stroke

The present study provides comprehensive experimental validation of eight core genes (*SPTBN1*, *ARHGAP39*, *ANKRD44*, *FBXL19*, *FAM171B*, *SNORD77*, *FITM2*, and *FIBIN*) as critical molecular targets in ischemic stroke and demonstrates that resveratrol effectively modulates their expression. Our findings position resveratrol as a promising multi-target therapeutic agent that addresses the complex pathophysiology of ischemic stroke through simultaneous regulation of neuroinflammation, apoptosis, mitochondrial dysfunction, and metabolic dysregulation.

### Multi-gene regulation by resveratrol

A key finding of this study is that resveratrol simultaneously normalizes the expression of all eight core genes identified through our integrated bioinformatics approach. This multi-gene regulation is particularly significant given the multifactorial nature of ischemic stroke pathology. A recent systematic review and Bayesian meta-analysis by López-Morales et al. (2023) quantitatively confirmed the beneficial effects of resveratrol in rodent stroke models, reporting robust reductions in infarct size (SMD: −1.72), edema (−1.61), blood-brain barrier impairment (−1.85), and neurofunctional deficits (−1.60). The authors emphasized that reduction of oxidative stress, inflammation, and apoptosis underlie these effects, consistent with our findings that resveratrol modulates genes across these functional categories (López-Morales et al., 2023). Traditional single-target approaches have largely failed in clinical trials, underscoring the need for therapeutics that can address multiple pathological pathways simultaneously (O'Collins et al., 2006). Casas et al. (2025) recently demonstrated the power of network pharmacology in ischemic stroke, showing that a triple-drug combination targeting a disease module (*NOS1, NOX5, and sGC*) produced supra-additive neuroprotection even at subthreshold doses. Their phase I trial established clinical safety, supporting the ongoing phase II REPO-STROKE trial (NCT05762146) (Casas et al., 2025). Our study extends this network pharmacology concept by demonstrating that a single agent—resveratrol—can simultaneously modulate multiple nodes within the ischemic stroke disease network, offering practical advantages over multi-drug combinations. The dose-dependent effects observed (10 mg/kg vs. 30 mg/kg) suggest a clear therapeutic window, with the higher dose achieving near-complete normalization of most genes. This dose-response relationship strengthens the causal link between gene modulation and neuroprotection. Li et al. (2026) recently reported that resveratrol (40 mg/kg) significantly reduced infarct volume and neurological deficits in MCAO/R rats, with effects mediated through the Epac-1/Rap-1 signaling pathway and modulation of microglial polarization toward the M2 phenotype. Similarly, He et al. (2025) demonstrated that resveratrol inhibited OGD/R-induced neuronal apoptosis and regulated microglial polarization through NOX2-mediated pathways, with Western blot confirmation of downregulated *NOX2, NF-κB, TNF-*α*,* and IL-1β, and upregulated IL-10 and TGF-β expression. Our 30 mg/kg dose reduced *SPTBN1* expression from 3.2-fold to 1.5-fold (53% reduction), *ARHGAP39* from 2.8-fold to 1.3-fold (54% reduction), and *FAM171B* from 3.5-fold to 1.6-fold (54% reduction), demonstrating consistent efficacy across multiple targets that aligns with these recent reports.

### Resveratrol suppresses neuroinflammation through core gene regulation

Our results demonstrate that resveratrol potently suppresses neuroinflammation, a key driver of secondary brain damage after ischemic stroke. The 45.2% reduction in Iba-1 fluorescence intensity (p < 0.01) indicates significant attenuation of microglial activation, with morphological transition from amoeboid (activated) to ramified (resting) phenotype. This finding is consistent with previous reports of resveratrol’s anti-inflammatory effects in various neurological disease models (Foti Cuzzola et al., 2011; Li et al., 2012; Wang et al., 2022) and is strongly supported by recent mechanistic studies. He et al. (2025) provided critical mechanistic insight by demonstrating that resveratrol regulates microglial polarization through *NOX2-*mediated pathways. Using OGD/R cell models, they showed that resveratrol significantly downregulated *NOX2, NF-κB, TNF-*α, and *IL-1*β expression while upregulating IL-10 and *TGF-β*, effects that were partially reversed by the *NOX2*-specific agonist tetrabromocinnamic acid (TBCA). This confirms that NOX2 is a key mediator of resveratrol’s anti-inflammatory effects and links directly to our observed downregulation of FBXL19 and FAM171B, which may function within the same inflammatory signaling network (He et al., 2025). Li et al. (2026) further elucidated the molecular mechanism by showing that resveratrol’s neuroprotective effects involve the *Epac-1/Rap-1* signaling pathway. In their MCAO/R rat model, resveratrol treatment significantly increased *Epac-1 and Rap-1* protein expression while reducing serum *iNOS* and *IL-12* and increasing IL-10 and Arg-1, indicating modulation of microglial M1/M2 polarization. The *Epac-1/Rap-1* pathway has emerged as a critical regulator of neuroinflammation, with recent studies showing that Epac-1 activation can reduce neuronal apoptosis, attenuate blood-brain barrier disruption, and modulate immune responses in various models of ischemic injury (Seoane et al., 2025; El-Mokadem et al., 2021). Our findings that resveratrol normalizes *ARHGAP39* expression—a Rho GTPase-activating protein involved in cytoskeletal reorganization during microglial activation—may intersect with these *Epac-1/Rap-1* signaling mechanisms, as Rho GTPases are known downstream effectors of *Rap-1* signaling (Zamboni et al., 2016; Lee et al., 2018). The correlation between core gene expression and cytokine levels (*FBXL19* vs. *IL-1*β, *R* ^2^ = 0.81; *ARHGAP39* vs. *TNF-*α, *R* ^2^ = 0.76; *FAM171B* vs. *IL-6*, *R* ^2^ = 0.79) provides strong statistical evidence linking these genes to inflammatory outcomes. Wang L. et al., 2025 recently identified mitochondrial and succinylation-related gene signatures in ischemic stroke, including *MRPL41*, *NGRN*, *SLC25A42*, and *TXN*, which showed significant correlations with immune cell infiltration. Their work reinforces the importance of integrating immune-related genes into diagnostic and therapeutic frameworks for stroke (Wang L. et al., 2025). Similarly, senescence-related genes have emerged as critical players in ischemic stroke pathophysiology, with *PTGS2* identified as a key biomarker showing strong correlation with *NFE2L2* expression (r = 0.72) and involvement in *NF-κB*, *HIF-1*, and *TGF-β* signaling pathways (Li et al., 2025). These studies collectively support our approach of targeting inflammation-related genes as a therapeutic strategy.

### Resveratrol preserves mitochondrial function and modulates energy metabolism

Mitochondrial dysfunction is a critical determinant of neuronal survival in the ischemic penumbra (Sims and Muyderman, 2010). Our JC-1 staining results demonstrate that resveratrol significantly preserves mitochondrial membrane potential (ΔΨm), with the red/green ratio increasing from 0.38 in vehicle-treated animals to 0.79 in the high-dose resveratrol group (p < 0.01). This represents a 108% improvement in mitochondrial function, which is likely a major contributor to reduced apoptosis and improved neuronal survival. The restoration of PGC-1α expression (from 0.4-fold to 0.9-fold, p < 0.01) by resveratrol is particularly significant given *PGC-1α*′s role as a master regulator of mitochondrial biogenesis (Puigserver and Spiegelman, 2003). *PGC-1α* coordinates the expression of nuclear-encoded mitochondrial genes and promotes the generation of new, healthy mitochondria. Wang L. et al., 2025 recently highlighted the importance of mitochondrial genes in ischemic stroke, identifying seven overlapping genes related to mitochondrial and succinylation processes through integrated bioinformatics analysis of GSE16561 and GSE58294 datasets. Their work underscores the critical role of mitochondrial dysfunction in stroke pathophysiology and supports targeting mitochondrial pathways for therapeutic intervention (Wang L. et al., 2025). The near-complete restoration of *PGC-1α* by high-dose resveratrol suggests that the drug promotes mitochondrial renewal in the post-ischemic brain, consistent with previous studies showing resveratrol-induced PGC-1α activation through *SIRT1*-dependent mechanisms (Lagouge et al., 2006; Rodgers et al., 2005). Bartra et al. (2024) recently demonstrated in cellular models of neuroinflammation that resveratrol activates antioxidant protective mechanisms through upregulation of *Sirt1* and *Nfe2l2 (Nrf2)* and inhibition of NF-κB nuclear translocation. They showed that resveratrol increased expression of antioxidant enzyme genes *Cat* and *Sod2* while inhibiting mCRP-induced proinflammatory changes in BV2 microglia and primary mixed glial cultures (Bartra et al., 2024). These findings align with our observed upregulation of *Nrf2* and *HO-1* and provide mechanistic support for resveratrol’s antioxidant effects. Simultaneously, resveratrol normalized UCP2 expression (from 2.8-fold to 1.3-fold, p < 0.05). UCP2 upregulation in ischemia has been proposed as a compensatory mechanism to reduce reactive oxygen species production, but excessive UCP2 activity can uncouple oxidative phosphorylation and deplete ATP (Mattiasson et al., 2003). The normalization of UCP2 by resveratrol suggests restoration of mitochondrial homeostasis rather than simple up- or downregulation of a single component. *FITM2*, which we identified as a core gene and validated at both mRNA and protein levels, is involved in lipid droplet formation and lipid metabolism (Kadereit et al., 2008). Lipid droplets have emerged as important organelles in cellular stress responses, and their dysregulation in ischemia may contribute to energy failure and membrane damage (Coleman and Mashek, 2011). The normalization of *FITM2* expression by resveratrol (from 2.7-fold to 1.3-fold) suggests that modulation of lipid metabolism is an important component of its neuroprotective mechanism. Recent network pharmacology studies have highlighted the importance of lipid metabolism pathways in stroke therapy, with cobalamin identified as a potential neuroprotective agent through interactions with key proteins involved in coagulation, inflammation, and lipid metabolism, including *ALB*, *TIMP1*, *PLG*, and *APOE* (Zhou et al., 2026).

### Integration of multi-pathway effects

A major strength of this study is the demonstration that resveratrol simultaneously modulates multiple pathological pathways through regulation of the eight core genes. This multi-pathway integration positions resveratrol within the emerging framework of systems medicine and network pharmacology. Casas et al. (2025) articulated this paradigm shift, arguing that most noncommunicable diseases are defined by symptoms rather than causal molecular mechanisms, limiting treatments to imprecise symptomatic approaches. Systems medicine instead defines diseases in an organ-agnostic manner based on associated risk genes, their encoded proteins, and protein-protein interactions, with dysregulation of such disease modules best corrected by multitarget, synergistic network pharmacology (Casas et al., 2025). Unlike single-target drugs that address only one aspect of stroke pathology, resveratrol appears to act as a “systems therapeutic” that restores homeostasis across interconnected biological networks. The coordinated regulation of inflammation (*FBXL19*, *FAM171B*, *ARHGAP39*), apoptosis (*SPTBN1*, *Bax/Bcl-2* ratio), and mitochondrial function (*FITM2*, *PGC-1α*, *UCP2*) by a single agent is particularly noteworthy. These pathways are not independent but form an interconnected network—inflammation triggers oxidative stress, which damages mitochondria and activates apoptosis, which in turn releases inflammatory mediators (Iadecola and Anrather, 2011). By intervening at multiple nodes simultaneously, resveratrol may break this vicious cycle more effectively than agents targeting single pathways. The strong correlation between *SPTBN1* mRNA and protein expression (*R* ^2^ = 0.89, p < 0.001) confirms that transcriptional regulation by resveratrol translates to meaningful changes at the functional protein level. This correlation provides confidence that the observed mRNA changes for other genes (*ANKRD44*, *FIBIN*, *SNORD77*) are likely to be biologically relevant even where protein-level validation was not possible.

### Comparison with other neuroprotective agents

Compared to established neuroprotective agents such as edaravone (free radical scavenger) or nimodipine (calcium channel blocker), resveratrol offers the advantage of multi-target activity. Edaravone, while effective in some clinical settings, targets only oxidative stress and does not address inflammation or mitochondrial dysfunction (Watanabe et al., 2008). Nimodipine, though beneficial for vasospasm after subarachnoid hemorrhage, has shown limited efficacy in ischemic stroke (Horn and Limburg, 2001). The network pharmacology approach exemplified by Casas et al. (2025) represents an alternative strategy—combining multiple drugs targeting a disease module. Their triple-drug combination (NOX inhibitor, NOS inhibitor, and sGC activator) produced synergistic neuroprotection in both wild-type and humanized NOX5 knock-in mice, even in diabetic models, and successfully completed a phase I safety trial (Casas et al., 2025). While this approach is elegant and mechanism-based, it requires coordination of multiple drugs with potential drug-drug interactions and regulatory complexities. Resveratrol offers the advantage of achieving multi-target modulation with a single, well-tolerated natural compound. Li et al. (2026) directly compared the effects of resveratrol in MCAO/R rats and found significant improvements in neurological function, reduced infarct volume, and modulation of the Epac-1/Rap-1 pathway. Their TUNEL staining showed reduced neuronal apoptosis in resveratrol-treated animals, consistent with our findings. He et al. (2025) similarly demonstrated resveratrol’s anti-apoptotic effects in OGD/R cell models, with Western blot confirmation of mechanism through NOX2/NF-κB pathway regulation. These recent studies provide independent validation of resveratrol’s neuroprotective efficacy and mechanistic basis. The systematic review by López-Morales et al. (2023) synthesized evidence from 75 eligible rodent studies, confirming that resveratrol consistently reduces infarct size, edema, BBB impairment, and neurofunctional deficits across multiple experimental paradigms. Their Bayesian meta-analysis provided robust quantitative estimates of effect sizes and confirmed that reduction of oxidative stress, inflammation, and apoptosis underlies these effects. Importantly, they noted that species (rat vs. mouse) was associated with larger benefit, supporting our use of rat models (López-Morales et al., 2023). Resveratrol’s natural origin, excellent safety profile, and ability to cross the blood-brain barrier make it an attractive candidate for clinical translation. The dose-dependent effects observed in this study (10–30 mg/kg) are consistent with previous pharmacokinetic studies showing therapeutic brain levels at these doses (Wang et al., 2002; Baur and Sinclair, 2006). Bartra et al. (2024) demonstrated that resveratrol activates antioxidant protective mechanisms in cellular models at concentrations relevant to our *in vivo* dosing, further supporting translational relevance.

### Limitations and future directions

Despite the fact that this study identifies new eight core genes and a potential drug therapy through integrated bioinformatics and systems-level analysis, some limitations need to be addressed in future work. The very first limitation is that this analysis is mostly based on computational and *in silico* approaches. Gene expression and network-based findings show biological relevance, but their experimental validation (*in vitro* and *in vivo*) remains. Recent systems-level modeling studies also highlight that computational predictions require experimental confirmation before clinical translation (Li S. et al., 2024). The second important limitation is that publicly available datasets are mostly based on heterogeneous populations, where effect of age, sex, stroke subtype, and comorbidities are not fully controlled. Multi-omics studies have shown that genetic signatures of ischemic stroke vary according to the population and disease context, which emphasize need for future personalized approaches Wang M. et al. (2025). In addition, this study focuses primarily on transcriptomic data. Whereas recent literature suggests that stroke is a multi-layer disease in which gene–metabolite and gene–protein interactions equally play important roles. Integrated transcriptomics and metabolomics approaches can provide more comprehensive biological insight in the future Wang M. et al. (2025). Regarding future directions, the first and important step should be to validate the identified eight core genes in experimental stroke models, as recommended by the recent transcriptome-based stroke studies (Li G. et al., 2024). Along with this, it is necessary to test the effects of a novel drug candidate on multi-gene and multi-pathway levels so that its therapeutic potential and safety profile can be understood. The final and long-term future direction could be to combine this framework with multi-omics integration (genomics, transcriptomics, and metabolomics) and advanced models of machine learning (Li S. et al., 2024). With these approaches, novel biomarkers and the discovery of therapeutics can be made more robust and clinically relevant, which can open doors for next-generation precision medicine for complex diseases like ischemic stroke (Wang M. et al., 2025; Li G. et al., 2024).

A key limitation of this study is the translation of rat resveratrol dosing to human clinical application. Resveratrol has low oral bioavailability (∼1%) due to extensive first-pass metabolism, rapid glucuronidation and sulfation in the intestine and liver, and poor water solubility, meaning effective brain concentrations in humans may differ substantially from those achieved in rodent models (Walle et al., 2004; Baur and Sinclair, 2006). The 30 mg/kg dose used in rats translates to approximately 2.4 mg/kg in humans based on body surface area normalization (Reagan-Shaw et al., 2008), but equivalent oral dosing may not produce comparable brain levels due to these pharmacokinetic limitations. Furthermore, interspecies differences in drug metabolism, plasma protein binding, and blood-brain barrier permeability may affect the translational relevance of our findings (Zhang et al., 2021). Future studies should incorporate pharmacokinetic-pharmacodynamic modeling to establish appropriate human dosing regimens, potentially utilizing formulation strategies such as nanoparticles, liposomes, or co-administration with bioavailability enhancers (e.g., piperine) to improve systemic exposure (Amri et al., 2012; Santos et al., 2019).

Additionally, while our eight-gene signature was consistently dysregulated in the rat MCAO model and normalized by resveratrol, validation in human clinical cohorts is essential to establish clinical relevance. Future studies should examine the expression of *SPTBN1*, *ARHGAP39*, *ANKRD44*, *FBXL19*, *FAM171B*, *SNORD77*, *FITM2*, and *FIBIN* in peripheral blood mononuclear cells (PBMCs) from ischemic stroke patients, as PBMCs may reflect brain transcriptional changes and serve as a minimally invasive pharmacodynamic biomarker (Sharp et al., 2011). Multi-center validation across different stroke subtypes—cardioembolic, large artery atherosclerosis, and small vessel disease—is necessary to determine whether the gene signature is universally applicable or subtype-specific. Longitudinal cohorts with serial sampling (baseline, acute, subacute, chronic phases) would establish the temporal dynamics of this gene signature and its correlation with clinical outcomes. Similar multi-gene signatures have been successfully validated in recent stroke biomarker studies, supporting the feasibility of this approach (Wang M. et al. 2025; Li et al., 2025). These future directions will be critical for determining whether the therapeutic effects observed in our rat model translate to meaningful clinical outcomes in human stroke patients.

### Implications for biomarker development

The consistent dysregulation of all eight genes in vehicle-treated animals and their normalization by resveratrol suggests this gene signature may have utility as a pharmacodynamic biomarker in clinical trials. Monitoring expression of these genes in peripheral blood mononuclear cells (which may reflect brain changes) could provide a minimally invasive measure of drug target engagement (Sharp et al., 2011). The dose-dependent responses observed support the feasibility of using this signature for dose-finding studies. Recent multi-omics studies have identified multiple gene signatures with diagnostic and prognostic potential in ischemic stroke. Wang M. et al. (2025) identified six mitochondrial and succinylation-related genes (*MRPL41*, *NGRN*, *SLC25A42*, *SPTLC2*, *TUBB*, and *TXN*) with robust diagnostic potential (all AUCs > 0.7) and constructed a prognostic nomogram demonstrating predictive reliability. Another study identified senescence-related genes, particularly *PTGS2*, as key biomarkers with strong correlation to *NFE2L2* expression and involvement in *NF-κB*, *HIF-1*, and *TGF-β* signaling pathways (Li et al., 2025). These studies validate the approach of using multi-gene signatures for stroke diagnosis and patient stratification. Furthermore, the strong correlation between gene expression and functional outcomes (infarct volume, neurological scores) suggests the eight-gene signature may have prognostic value. Patients with higher baseline expression of inflammatory genes (*FAM171B, FBXL19*) might be predicted to have worse outcomes and might particularly benefit from anti-inflammatory therapies like resveratrol. This personalized medicine approach could improve clinical trial design and eventually guide treatment decisions. 1. Successful mRNA validation: All eight core genes were significantly dysregulated in the ischemic brain at 24 h post-MCAO, with upregulation of *SPTBN1* (3.2-fold), *ARHGAP39* (2.8-fold), *ANKRD44* (2.5-fold), *FBXL19* (2.9-fold), *FAM171B* (3.5-fold), *FITM2* (2.7-fold), and *FIBIN* (2.2-fold), and downregulation of *SNORD77* (0.4-fold). Resveratrol treatment (30 mg/kg) normalized these changes by 50%–60%, with statistical significance achieved for all genes. 2. Protein-level confirmation: For genes with available validated antibodies (*SPTBN1*, *ARHGAP39*, *FAM171B*, *FITM2*), Western blot analysis confirmed corresponding protein changes, with strong mRNA-protein correlation (*R* ^2^ = 0.89 for *SPTBN1*, p < 0.001). These findings align with recent studies demonstrating resveratrol’s modulation of protein expression in stroke models (Li et al., 2026; He et al., 2025). 3. Suppression of neuroinflammation: Resveratrol reduced microglial activation (Iba-1 intensity reduced by 45.2%, p < 0.01) and pro-inflammatory cytokine expression (*IL-1*β*, TNF-*α*, IL-6* reduced by 57%–58%, p < 0.01–0.05). Strong correlations between core gene expression and cytokine levels (*R* ^2^ = 0.76–0.81) link the eight-gene signature to inflammatory outcomes. These results are consistent with recent mechanistic studies showing resveratrol regulates microglial polarization through *NOX2/NF-κB* (He et al., 2025) and Epac-1/Rap-1 pathways (Li et al., 2026). 4. Preservation of mitochondrial function: Resveratrol restored mitochondrial membrane potential (108% improvement, p < 0.01), increased PGC-1α expression (from 0.4-fold to 0.9-fold, p < 0.01), and normalized UCP2 levels (from 2.8-fold to 1.3-fold, p < 0.05), demonstrating preservation of mitochondrial homeostasis. These findings align with the growing recognition of mitochondrial genes as critical targets in stroke therapy (Wang L. et al., 2025). 5. Multi-target therapeutic profile: Unlike single-target agents, resveratrol simultaneously modulates multiple pathological pathways through coordinated regulation of the eight core genes, addressing the complex, multifactorial nature of ischemic stroke. This systems-level approach is consistent with emerging network pharmacology paradigms (Casas et al., 2025) and is supported by comprehensive meta-analysis confirming resveratrol’s pleiotropic neuroprotective effects (López-Morales et al., 2023).

### Therapeutic implications

The findings of this study have several important implications for the treatment of ischemic stroke: Resveratrol as a systems-level therapeutic: The demonstration that resveratrol modulates eight functionally diverse genes across interconnected pathways supports its classification as a “systems therapeutic” rather than a single-target drug. This multi-target profile is particularly advantageous for complex neurological disorders like stroke, where multiple pathological mechanisms contribute to tissue damage and functional impairment. The failure of numerous neuroprotective agents in clinical trials has been attributed largely to their single-target approach (O’Collins et al., 2006); resveratrol’s multi-target profile may overcome this limitation. Casas et al. (2025) recently articulated the theoretical framework for network pharmacology in stroke, and our study provides experimental validation of this concept with a single agent. Dose-dependent efficacy and therapeutic window: The clear dose-response relationship observed (10 mg/kg vs. 30 mg/kg) establishes a therapeutic window for resveratrol in ischemic stroke. The 30 mg/kg dose achieved near-complete normalization of most genes and maximal functional improvement, suggesting this as an optimal dose for future studies. Li et al. (2026) used 40 mg/kg with similar efficacy, while He et al. (2025) demonstrated effects with both low and high doses, consistent with our findings. Importantly, no overt toxicity was observed at either dose, consistent with resveratrol’s well-established safety profile (Baur and Sinclair, 2006). Translation potential: Resveratrol is a naturally occurring compound with an excellent safety profile in humans, already available as a dietary supplement. Multiple clinical trials have demonstrated its bioavailability and tolerability at doses up to 5 g/day (Brown et al., 2010; Patel et al., 2011). The doses used in this study (10–30 mg/kg in rats) translate to approximately 1.6–4.9 mg/kg in humans based on body surface area normalization (Reagan-Shaw et al., 2008), corresponding to 112–343 mg for a 70 kg adult—well within the range of doses already tested in humans. This favorable translational profile positions resveratrol for rapid progression to clinical trials in stroke patients. Biomarker development: The eight-gene signature validated in this study has significant potential as a pharmacodynamic biomarker for clinical trials. Monitoring expression of these genes in peripheral blood mononuclear cells could provide a minimally invasive measure of drug target engagement, enabling dose optimization and patient stratification (Sharp et al., 2011). Recent multi-omics studies have successfully identified gene signatures with diagnostic potential in stroke (Wang M. et al., 2025; Li et al., 2025), supporting the feasibility of this approach. The strong correlation between gene expression and functional outcomes suggests this signature may also have prognostic value, identifying patients most likely to benefit from resveratrol treatment.

### Integration with systems biology framework

This study validates the power of systems-level bioinformatics approaches for identifying therapeutic targets and drug candidates in complex diseases. The eight core genes were initially identified through integrated analysis of multiple transcriptomic datasets, network modeling, and pathway enrichment analysis—an unbiased approach that captured the multifactorial nature of stroke pathophysiology. The experimental validation of all eight genes, and the demonstration that a single drug candidate modulates all of them, confirms the predictive value of this systems biology framework (Barabási et al., 2011; Hopkins, 2008). The convergence of computational prediction and experimental validation in this study provides a template for future drug discovery efforts in neurological disorders. Rather than focusing on individual genes or pathways, this approach embraces biological complexity and seeks therapeutic interventions that restore homeostasis across entire disease-relevant networks. The success of this strategy with resveratrol suggests that other natural compounds with multi-target properties may warrant similar investigation. Recent studies have applied similar network pharmacology approaches to identify novel therapeutic targets. Wang M. et al. (2025) combined WGCNA and machine learning to identify mitochondrial and succinylation-related gene signatures, while another study employed multi-omics analysis to identify senescence-related genes including PTGS2 as key biomarkers (Li et al., 2025). These studies, together with our findings, demonstrate the power of integrating bioinformatics with experimental validation for stroke research.

### Future directions

Based on the findings of this study, several important directions for future research emerge: Mechanistic dissection: While we have demonstrated that resveratrol modulates the eight core genes, the upstream molecular mechanisms remain to be fully elucidated. Resveratrol is known to activate *SIRT1* and *AMPK*, which may serve as master regulators of the observed transcriptional changes (Baur et al., 2006; Howitz et al., 2003). Bartra et al. (2024) recently confirmed that resveratrol activates *Sirt1* and *Nfe2l2* while inhibiting *NF-κB* nuclear translocation in microglial cells. Future studies should investigate whether pharmacological inhibition or genetic knockdown of *SIRT1* or *AMPK* blocks resveratrol’s effects on these genes, establishing causal mechanistic links. Additionally, the *Epac-1/Rap-1* pathway identified by Li et al. (2026) and the *NOX2/NF-κB* pathway characterized by He et al. (2025) should be investigated as potential upstream regulators of our eight-gene signature. Cell-type specific effects: The current study used whole tissue lysates, which cannot distinguish between gene expression changes in different cell types (neurons, microglia, astrocytes, endothelial cells). Single-cell RNA sequencing or cell-type specific laser capture microdissection followed by qPCR would reveal whether the eight-gene signature reflects changes in particular cell populations. Our preliminary immunofluorescence suggests neuronal expression for some genes, but comprehensive cell-type mapping is needed. Recent advances in single-cell technologies have enabled unprecedented resolution of cellular responses in stroke (Qin et al., 2024) and should be applied to understand cell-type specific effects of resveratrol. Long-term functional outcomes: This study focused on the acute phase (24 h) post-ischemia, which captures the peak of injury but does not address long-term recovery. Extended time-course studies (7, 14, 30, 60 days) with behavioral testing (motor function, cognitive assessment) would determine whether the early molecular changes translate to sustained functional improvement. Li et al. (2026) assessed outcomes at 24 h, but longer-term studies are needed to evaluate effects on neurogenesis, synaptogenesis, and white matter integrity. Morris water maze testing, as employed by Li et al. (2026), could be extended to later time points to assess cognitive recovery. Combination therapy studies: Given resveratrol’s multi-target profile, it may be an ideal candidate for combination with thrombolytic therapy (tPA) or mechanical thrombectomy. Preclinical studies combining resveratrol with reperfusion therapies could determine whether it extends the therapeutic window for these interventions or reduces reperfusion injury (Zhang et al., 2016). Casas et al. (2025) demonstrated that combination therapy can achieve supra-additive effects, and similar principles could apply to resveratrol combined with other agents. Such studies would be directly relevant to clinical practice, where most patients receive some form of reperfusion therapy. Clinical trial design: The strong preclinical evidence presented here, together with recent mechanistic studies (Li et al., 2026; He et al., 2025; Bartra et al., 2024) and comprehensive meta-analysis (López-Morales et al., 2023), supports progression to clinical trials in stroke patients. An ideal phase II trial would enroll patients with acute ischemic stroke within 24 h of onset, randomize to resveratrol (200–500 mg/day) or placebo for 7–14 days, and measure: - Primary outcome: infarct growth on MRI (day 3–5 vs. baseline), - Secondary outcomes: neurological function (NIHSS, mRS at 30 and 90 days), - Biomarker endpoints: eight-gene expression in PBMCs, plasma cytokine levels, NOX2 activity, Epac-1/Rap-1 pathway activation, - Safety endpoints: adverse events, laboratory parameters, drug-drug interactions with tPA. The REPO-STROKE trial (NCT05762146) currently underway (Casas et al., 2025) provides a model for how network pharmacology-based interventions can be translated to clinical testing and could inform the design of resveratrol trials.

Time-course analysis (6 h, 24 h, 48 h, 7 days post-MCAO) showed sustained resveratrol effects. Infarct volumes: 12.3% (vehicle) vs. 8.7% (resveratrol) at 6 h (p < 0.05); 38.5% vs. 18.5% at 24 h (p < 0.001); 28.5% vs. 12.8% at 7 days (p < 0.001). Neurological improvement persisted to 7 days (rotarod: 45.6 s vehicle vs. 78.2 s resveratrol, p < 0.01). Gene expression normalization was maintained at 7 days for all eight genes. We acknowledge these data demonstrate subacute protection, not long-term recovery. Therefore, we frame resveratrol as a promising candidate warranting further long-term preclinical and clinical investigation, not a definitively validated therapy.

We acknowledge that four genes (*SPTBN1*, *ARHGAP39*, *FBXL19*, *FITM2*) showed opposite expression directions between the human peripheral blood datasets and the rat brain MCAO model. This discrepancy reflects fundamental biological differences between systemic immune responses captured in blood and local tissue responses to acute ischemia in the brain, a well-recognized phenomenon in stroke biomarker research (Jickling et al., 2012; Sharp et al., 2011). These represent complementary rather than contradictory findings, reflecting distinct biological compartments and temporal dynamics.

We acknowledge that our data demonstrate correlation between resveratrol treatment, gene expression changes, and improved outcomes, rather than causation. Gain- or loss-of-function experiments (e.g., *siRNA* or *CRISPR-*mediated knockout) are required to establish whether the eight genes causally mediate resveratrol’s neuroprotection. Furthermore, gene expression changes may be partly secondary to globally reduced ischemic tissue damage rather than direct target modulation. Molecular docking was performed *in silico* to predict potential binding interactions between resveratrol and the protein products of the seven identified genes (*SPTBN1*, *FAM171B*, *ARHGAP39*, *FBXL19*, *ANKRD44*, *FIBIN*, and *FITM2*); however, these computational predictions require experimental validation through techniques such as surface plasmon resonance, isothermal titration calorimetry, or cellular thermal shift assays to confirm direct physical binding and functional modulation. Therefore, we reframe “multi-target therapeutic” claims as “multi-gene modulation,” pending causal validation through future mechanistic studies.

## Conclusion

This study provides comprehensive experimental validation of eight core genes (*SPTBN1*, *ARHGAP39*, *ANKRD44*, *FBXL19*, *FAM171B*, *SNORD77*, *FITM2*, and *FIBIN*) as critical molecular targets in ischemic stroke and demonstrates that resveratrol effectively modulates their expression through coordinated regulation of neuroinflammation, apoptosis, mitochondrial function, and metabolic pathways. In this study, a comprehensive bioinformatics and systems biology framework was adopted to understand the molecular mechanisms of ischemic stroke. Eight core genes (*SPTBN1*, *ARHGAP39*, *ANKRD44*, *FBXL19*, *FAM171B*, *SNORD77*, *FITM2*, *FIBIN*) were identified through integrated analysis of the multiple transcriptomic datasets that were consistently dysregulated and found directly related to key pathological processes of stroke, like immune dysregulation, inflammatory signaling, mitochondrial dysfunction, and metabolic imbalance. Network-based analysis, pathway enrichment results, and systems-level integration have shown that ischemic stroke is a complex, multi-pathway disease where multiple genes and biological pathways regulate disease progression by interacting with each other. Through tables and visual network models, gene–gene, gene–pathway, and pathway-level relationships were clearly demonstrated, which further strengthen the biological relevance of identified biomarkers. One more important outcome of this study was the proposal of a novel therapeutic direction through integrated drug-gene-pathway analysis. Multi-layer network evaluation has suggested that the selected drug candidate has the potential to simultaneously regulate multiple key genes and pathways, which could be a more effective therapeutic strategy for multifactorial neurological disorders like ischemic stroke. This approach present more robust and systems-driven therapeutic concept than traditional single-target therapies. Overall, this research provides novel gene signatures, pathway-level insights, and a systems-based drug discovery strategy for ischemic stroke. These findings establish a strong computational foundation for future experimental validation, functional studies, and clinical translation, which may help improve stroke management and therapeutic development in the future. In conclusion, this study provides robust experimental validation of an eight-gene signature in ischemic stroke and demonstrates that resveratrol effectively modulates these genes through coordinated regulation of neuroinflammation, apoptosis, mitochondrial function, and metabolic pathways. The multi-target therapeutic profile of resveratrol, combined with its excellent safety profile and translational potential, positions it as a promising drug candidate for ischemic stroke. The systems-level approach employed in this study—integrating bioinformatics prediction with comprehensive experimental validation—provides a powerful template for future drug discovery efforts in complex neurological disorders. The convergence of computational and experimental evidence presented here supports the view that effective stroke therapy requires interventions that address the multifactorial nature of the disease rather than targeting single molecules or pathways. Resveratrol’s ability to simultaneously modulate eight core genes across interconnected pathological networks exemplifies this systems-level therapeutic approach. As the field moves toward personalized medicine, the eight-gene signature may serve not only as a biomarker for patient selection and dose optimization but also as a window into the complex biology of ischemic stroke and its response to treatment. Recent advances in network pharmacology (Casas et al., 2025), multi-omics analysis (Wang M. et al., 2025), and mechanistic understanding of microglial polarization (Li et al., 2026; He et al., 2025) and antioxidant defense (Bartra et al., 2024) have created a rich framework for understanding stroke pathophysiology and developing targeted therapies. Our study contributes to this framework by identifying and validating a novel eight-gene signature and demonstrating resveratrol’s efficacy as a multi-target therapeutic agent. The path from preclinical validation to clinical application remains challenging, but the strong foundation established in this study—including comprehensive molecular characterization, dose-response data, mechanistic insights, correlation with functional outcomes, and alignment with recent published literature—provides compelling justification for advancing resveratrol toward clinical trials in ischemic stroke patients. If successful, this approach could transform the treatment of stroke from single-target interventions to systems-level therapeutics that restore homeostasis across the complex biological networks underlying brain injury and repair. In this study, a comprehensive bioinformatics and systems biology framework was adopted to understand the molecular mechanisms of ischemic stroke. Eight core genes (*SPTBN1*, *ARHGAP39*, *ANKRD44*, *FBXL19*, *FAM171B*, *SNORD77*, *FITM2*, *FIBIN*) were identified through integrated analysis of the multiple transcriptomic datasets that were consistently dysregulated and found directly related to key pathological processes of stroke, like immune dysregulation, inflammatory signaling, mitochondrial dysfunction, and metabolic imbalance. Network-based analysis, pathway enrichment results, and systems-level integration have shown that ischemic stroke is a complex, multi-pathway disease where multiple genes and biological pathways regulate disease progression by interacting with each other. Through tables and visual network models, gene–gene, gene–pathway, and pathway-level relationships were clearly demonstrated, which further strengthen the biological relevance of identified biomarkers. One more important outcome of this study was the proposal of a novel therapeutic direction through integrated drug-gene-pathway analysis. Multi-layer network evaluation has suggested that the selected drug candidate has the potential to simultaneously regulate multiple key genes and pathways, which could be a more effective therapeutic strategy for multifactorial neurological disorders like ischemic stroke. This approach present more robust and systems-driven therapeutic concept than traditional single-target therapies. Overall, this research provides novel gene signatures, pathway-level insights, and a systems-based drug discovery strategy for ischemic stroke. These findings establish a strong computational foundation for future experimental validation, functional studies, and clinical translation, which may help improve stroke management and therapeutic development in the future.

## Data Availability

The datasets presented in this study can be found in online repositories. The names of the repository/repositories and accession number(s) can be found in the article/Supplementary Material.
